# Are diabetes self-management programmes for the general diabetes population effective for people with severe mental illness?: a systematic review

**DOI:** 10.1186/s12888-020-02779-7

**Published:** 2020-07-25

**Authors:** Anne Coxon, Hayley McBain, Neli Pavlova, Hannah Rowlands, Kathleen Mulligan

**Affiliations:** 1 Centre for Health Services Research, School of Health Sciences, City, University of London, Northampton Square, London, EC1V 0HB UK; 2 Community Health Newham, East London NHS Foundation Trust, Trust Headquarters, 9 Alie Street, London, E1 8DE UK

**Keywords:** Diabetes self-management education, DSME, Severe mental illness, SMI, Type 2 diabetes

## Abstract

**Background:**

Diabetes self-management education programmes are effective in improving health outcomes in the general population with diabetes. However, it is not known if these programmes include people who also have a severe mental illness (SMI) and, if so, what their outcomes are. The aim of this review was to examine if evaluations of diabetes self-management education programmes included people with SMI, and if so, whether the interventions were beneficial for this population.

**Methods:**

The inclusion criteria for this systematic review, defined by PICOS criteria, were: Population - Adults with type 2 diabetes; Intervention - self-management education programme; Comparator – another active intervention or usual care; Outcomes of interest – inclusion of people with SMI and the clinical, behavioural and psychosocial outcomes in this population; Study design - randomised controlled trials.

The following bibliographic databases were searched from January 2004 to April 2018: Cochrane Library, Medline, Embase, PsychINFO, Allied and Complimentary Medicine Database, Health Technology Assessment, NHS Economic Evaluations Database and CINAHL. Data were extracted on study characteristics, inclusion and exclusion criteria, participant and intervention characteristics, number of participants with SMI, and outcomes for people with SMI, if reported. Authors were contacted by email for missing data.

**Results:**

A total of 410 trials were included. At least 42% of trials did not recruit any participants with SMI. Only nine confirmed inclusion of participants with SMI, of which six provided data on the number recruited. These six trials recruited a total of 1009 participants, of whom 31 (3.1%) had SMI. It was not possible to assess intervention effectiveness for people with SMI as none of the trials reported outcomes for these participants.

**Conclusions:**

This systematic review confirms that people with SMI are often excluded from trials of diabetes self-management education, resulting in a lack of an evidence base on which to base treatment paths for this vulnerable population. It cannot be assumed that programmes developed for the general diabetes population meet the needs of people with SMI. Future research needs to examine if and how these programmes could be adapted for people with SMI or if new programmes are required.

## Background

The estimated prevalence of diabetes mellitus in people with psychosis is 13%, which is between two and five times higher than the general population [1]. Several factors are thought to contribute to this increased risk, including the effects of anti-psychotic medications, pathophysiology of SMI and lifestyle factors such as poor diet, obesity and physical inactivity [2]. Among people with diabetes mellitus, the risk of acute complications and mortality is also greater in those with SMI [3, 4].

Self-management of type 2 diabetes mellitus (T2DM) is complex, and to achieve this successfully, diabetes clinical guidelines [5–9] recommend structured education for all patients. A number of systematic reviews have reported the positive effects of diabetes self-management education (DSME) programmes [10–12], such as better glycaemic control, greater diabetes knowledge and self-management skills, and higher self-efficacy, but it is unclear if people with SMI also experience these benefits. A recent Cochrane review of DSME specifically for people with SMI [12], conducted by members of the current authorship team, identified only one intervention [13], Diabetes Awareness and Rehabilitation Training (DART), which was evaluated in 64 people aged over 40 with T2DM and either schizophrenia or schizoaffective disorder. DART used adapted materials and reinforced behaviour change, to help overcome impaired motivation and insight. At the end of the 24-week trial, the DART group experienced a greater reduction in weight, body mass index (BMI), waist circumference and plasma triglycerides than controls, and had significantly increased their diabetes knowledge, diabetes self-efficacy and self-reported physical activity. The effects on participants’ BMI, waist circumference and diabetes knowledge were maintained at 6-month follow-up [14]. There were however no statistically significant changes in fasting glucose or glycosylated haemoglobin (HbA1_C_) levels. This study indicates that where interventions are developed to address the particular needs of people with SMI and T2DM, positive lifestyle changes can be achieved. The DART programme has since been combined with the Life Goals Program [15] (an intervention that focuses on mental health but not diabetes) to form the Targeted Training in Illness Management (TTIM) intervention, which has been tested in an RCT with 200 individuals with SMI and type 2 diabetes [16]. This 12-week group programme resulted in significantly better mental health and diabetes knowledge in the intervention group but no group differences were found in diabetes self-management behaviour or HbA1c.

Given that neither intervention [13, 16] achieved a change in HbA1_c_, this suggests a need for further research to identify how best to optimise diabetes self-management for people with SMI to improve clinical as well as behavioural outcomes. The sparsity of evidence from SMI-specific DSME highlights that it is important to try to determine whether evidence of efficacy of DSME developed for the general diabetes population is also applicable to people with SMI. A rapid synthesis of the evidence on interventions supporting self-management [17] identified 179 unique randomised controlled trials (RCTs) of DSME in the general diabetes population. This plethora of data may provide insight into whether and how DSME programmes for the general population have been implemented for people with SMI, and if they have been successful.

This systematic review therefore sought to answer the question: Are DSME programmes for the general diabetes population effective for people with SMI?

## Method

Inclusion criteria, defined by Population, Intervention, Comparator, Outcome, and Study design (PICOS) [18] were:

- **P**opulation **-** Adults aged 18 or over and diagnosed with T2DM.
- **I**ntervention - Interventions that were targeted to improve the self-management of T2DM by providing structured education. This could include interventions that targeted diabetes self-management behaviours such as self-monitoring of blood glucose, medication adherence, foot care, diet or physical activity. Although self-management of SMI could be included, interventions that focused solely on the management of SMI without any diabetes education were excluded. Interventions could be delivered individually or in groups, in person or remotely e.g. telephone or online.
- **C**omparator – comparators were either another active intervention or usual care.
- **O**utcomes – outcomes of interest were: inclusion of people with SMI (defined as psychosis, schizophrenia, schizoaffective disorder, bipolar disorder, depression with psychotic features or personality disorder); number (%) of participants with SMI; plus clinical (HbA1c, body mass index, weight, blood pressure), behavioural (diabetes self-care behaviours such as blood glucose monitoring, medication adherence, diet and physical activity), and psychosocial (health-related quality of life, diabetes knowledge, self-efficacy) outcomes for participants with SMI.
- **S**tudy design - RCTs

Publications were excluded if they:

- included only participants with type 1 diabetes or gestational diabetes
- were written in languages other than English
- were published as conference abstracts, editorials, or letters
- had not undergone formal peer review.

Trials that recruited *only* people with T2DM and SMI were also excluded as the recent Cochrane review [12] had already reviewed these.

### Search strategy

A systematic literature search was undertaken in the following databases: Cochrane Library, Medline, Embase, PsychINFO, Allied and Complimentary Medicine Database, Health Technology Assessment, NHS Economic Evaluations Database and CINAHL from January 2004 to April 2018. The search included terms for diabetes, patient education/self-management and RCTs. The full list of terms is reported in Additional file 1.

### Study selection

Retrieved articles were imported into Reference Manager bibliographic software version 12 and duplicates removed. Titles and abstracts were independently screened against inclusion criteria by two reviewers (two of AC, KM, HR, NP, RS). Full texts of the remaining articles were then obtained and independently screened for inclusion by two reviewers (two of AC, KM, HR, NP, RS). Any disagreements were discussed with a third reviewer (KM or HM).

Data were extracted by three of the review team (AC, NP, HR) using an adapted Cochrane data extraction form [19], which included information on study characteristics, inclusion and exclusion criteria, participant characteristics, intervention characteristics, number included with SMI, and outcomes for people with SMI, if reported. If the article did not report this information on SMI, authors were contacted by email for missing data.

Risk of bias in those trials that reported inclusion of people with SMI was independently assessed by two authors (KM and HM) using the Cochrane Collaboration tool [20]. Trials were rated as low, high or unclear risk of bias across seven criteria: sequence generation; allocation concealment; blinding of participants or personnel; blinding of outcome assessors; incomplete outcome data; selective reporting and other bias. Any disagreements were discussed to achieve consensus. Potential publication bias was assessed by creating a funnel plot of effect estimates against their standard errors for the outcome of Hba1c and conducting Egger’s test for asymmetry of the funnel plot.

### Data analysis

Descriptive statistics were used to calculate the proportion of trials that included participants with SMI. Where trials reported outcomes for participants with SMI, we planned to assess treatment effects using a random-effects meta-analysis.

## Results

**Fig. 1:**
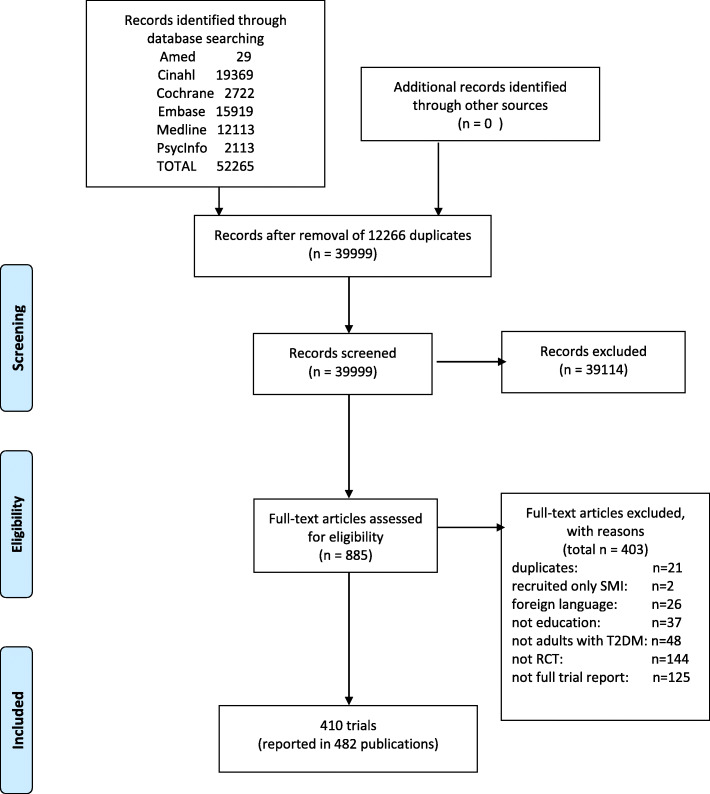
PRISMA flow diagram

From an initial 52,265 titles, 885 full texts were assessed for eligibility. A total of 410 RCTs, conducted in 53 different countries, were included in the review. (See PRISMA flowchart shown in Fig. 1). Studies conducted in the USA dominated (*n* = 134, 33%), followed by the UK (32, 8%), Iran (23, 5.6%) and China (20, 4.9%). Six trials (1.5%) were multinational.

Results for recruitment of people with SMI are reported in Table 1 and Additional file 2. Of 410 included trials, 110 (26.8%) listed SMI in the publication inclusion/exclusion criteria (of which 109 excluded SMI), we received information from authors on a further 123 (30%) but authors of 177 (43.2%) trials did not respond to our email requests for information.

**Table 1:** Number and % of RCTs that included or excluded participants with SMI

			TOTALS	
	N	%	*n*	%
**Included SMI**			**9**	2.2
*Authors provided data on numbers recruited but not effectiveness*	6	1.5		
*Authors did not provide data on numbers recruited or effectiveness*	3	0.7		
**Did not include SMI**			**172**	42.0
*Explicitly excluded SMI:*				
*Reported in paper*	109	26.6		
*Response from authors*	47	11.5		
*Not explicitly excluded but no-one with SMI recruited*	16	3.9		
**Unknown if SMI included**			**229**	55.9
*Authors did not collect data on SMI*	52	12.7		
*Authors did not respond to email request*	177	43.2		
TOTAL RCTs	410		**410**	

Of the 410 trials, at least 172 (42%) RCTs did not recruit any participants with SMI; this was 74% of the 233 trials on which we were able to obtain data. In 229 (55.9%) RCTs, it is unknown if people with SMI were recruited, either because the authors did not respond to our request for information (*n* = 177, 43.2%) or because the authors were unaware if they had recruited any participants with SMI as this data was not collected (*n* = 52, 12.7%).

Nine (2.2%) RCTs [21–29] confirmed inclusion of people with SMI following email requests; however, of these, only six [21–23, 25, 28, 29] provided data on the numbers recruited and none provided data on effectiveness of the interventions in their participants with SMI. The six trials that provided data recruited a total of 1009 participants with T2DM, of whom 31 (3.1%) had SMI. This is in line with population prevalence, in which approximately 2% of people with type 2 diabetes have SMI (approximately double that of the general population) [29, 30].

The six trials that provided data on participants with SMI were conducted in Brazil [23], New Zealand [28], Qatar [22], Slovenia [29] and the United States [21, 25]. The interventions they evaluated were varied. Mohamed et al. [22] trialled a culturally sensitive group intervention including education on diabetes, healthy eating and exercise, and included five participants with schizophrenia. An individual programme trialled in Brazil [23] evaluated education plus blood glucose monitoring and pharmacotherapy adjustment. This trial included one participant with bipolar disorder, two with depression with psychotic features, and one participant diagnosed with personality disorder. The DECIDE education and problem-solving training programme was evaluated in two trials with African-American populations [21, 25]. The first [21] compared intensive and condensed versions and included one participant with schizophrenia, two with bipolar disorder and one participant with other psychosis. The second [25] compared self-study, individual and group versions of DECIDE and recruited eight people with SMI, six with schizophrenia, one with bipolar disorder and one who had depression with psychotic features. Whitehead et al. 2017 [28] recruited one person with SMI (personality disorder) to their comparison of nurse-led education plus Acceptance and Commitment Therapy (ACT) with nurse-led education alone or usual care. Two different levels of intensity of multidisciplinary care plus education were compared in a trial [23] that recruited four participants with SMI - one with bipolar disorder, two who had depression with psychotic features and one with personality disorder. The final trial [29], which recruited six people with SMI (one with schizophrenia, one with schizoaffective disorder, three with bipolar disorder and one who had depression with psychotic features) evaluated telemedicine plus education compared with usual care. None of these interventions incorporated components that specifically targeted issues around managing SMI.

**Fig. 2:**
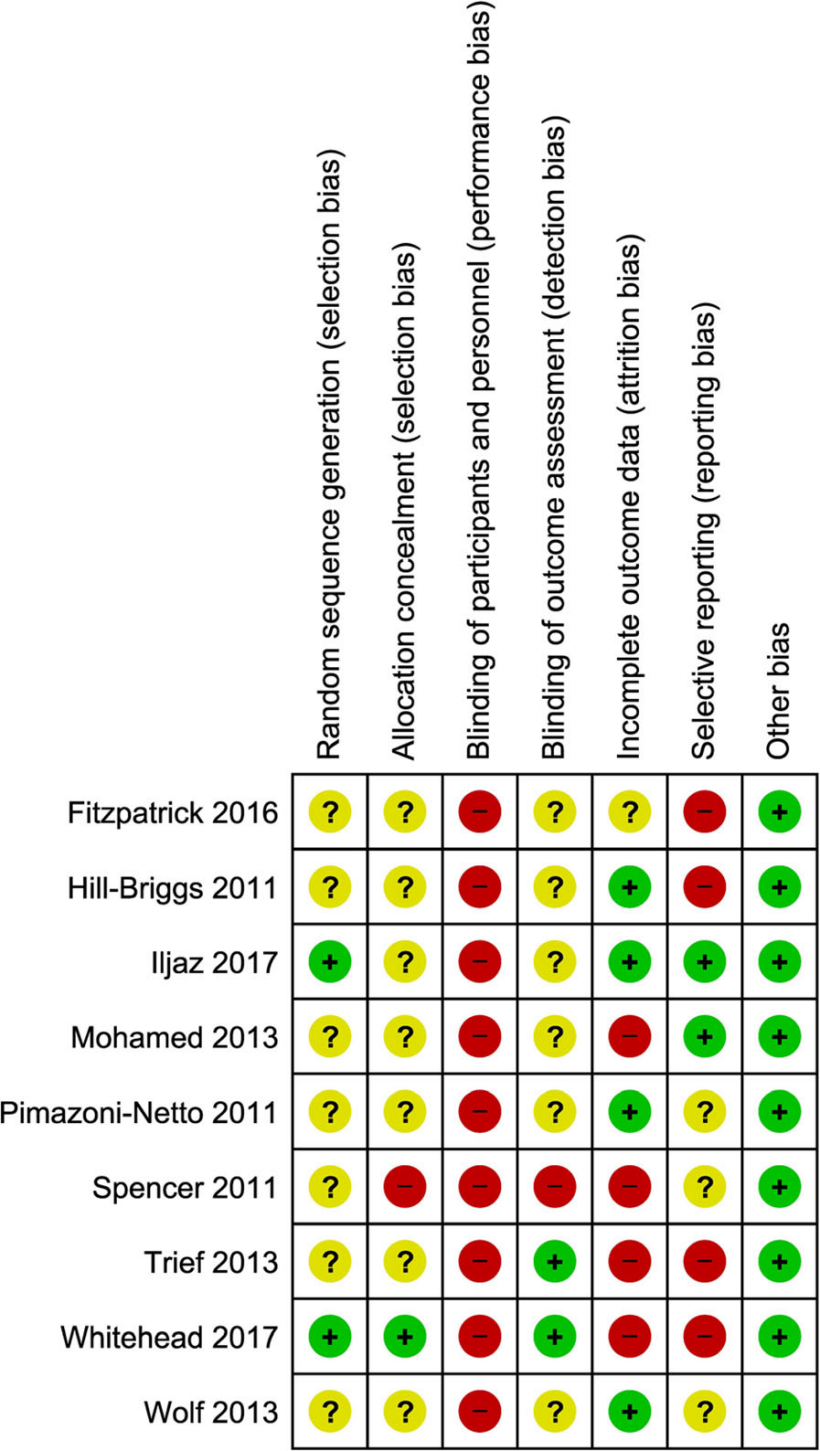
Risk of bias summary for trials that included participants with severe mental illness

**Fig. 3:**
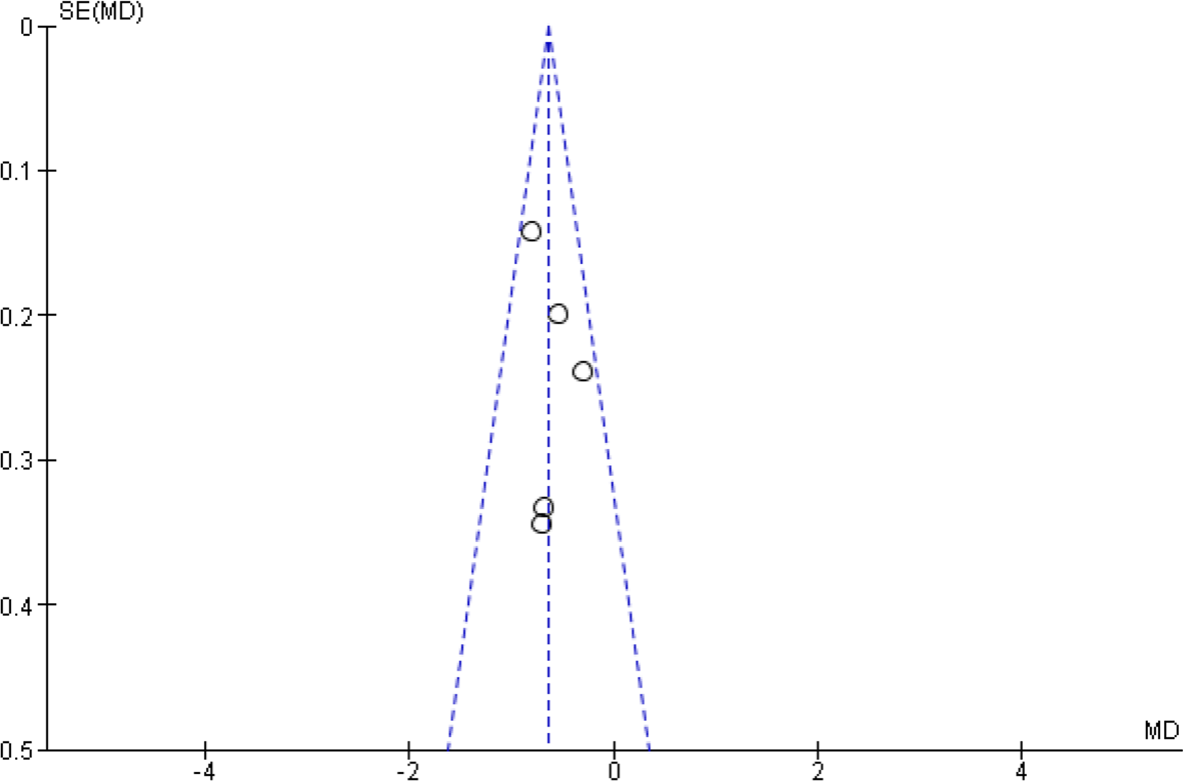
Funnel plot of estimates of effect on HbA1c

Assessment of Risk of Bias for the nine trials that included people with SMI is shown in Fig. 2. Risk of bias was mostly unclear for random sequence generation, allocation concealment and blinding of outcome assessment. All trials were rated high risk of bias for blinding of participants and personnel, which is unavoidable given the type of intervention. Risk of bias for incomplete outcomes data was rated as high in four trials, mostly because of differential dropout between trial arms. Selective reporting was rated as high or unclear risk of bias in most studies where they had not reported registration of the trial protocol and/or had not reported data in sufficient detail to enter into a meta-analysis/funnel plot. Only five trials reported sufficient data for the funnel plot, which is shown in Fig. 3. The result of the Egger’s test for asymmetry of the funnel plot was non-significant (β 0.54, SE 0.17 (95% CI 0.08–1.01), *p* = 0.47), which suggests a low risk of publication bias, however this finding should be interpreted with caution in view of the small number of included trials.

The trials did not report, or were unable to provide, the results for participants with SMI; we therefore did not conduct analysis of treatment effects in people with SMI for these trials.

## Discussion

The key findings of this review reveal that at least 42% of identified trials of DSME do not include any participants with SMI and only 2% had participants with a known diagnosis of SMI.

None of the interventions tested in these trials incorporated components that specifically targeted mental health issues that may affect self-management of diabetes and none of the trials reported data on effectiveness of the interventions for people with SMI.

Given the increased risk of T2DM in people with SMI and the large number of RCTs of DSME that have been conducted, their omission from trials of DSME is remarkable. However, our findings echo previous research [31], which has also found that people with SMI are often excluded from clinical trials. Of 400 highly cited trials across 20 common chronic health conditions published between 2002 and 2010, 55% of the papers that described diabetes research had psychiatric exclusion criteria [31]. Humphreys et al. argued that excluding this population results in knowledge gaps that may harm people with SMI when a treatment becomes generalised [31].

The recent Cochrane review [12] identified only one intervention [13] that had been developed and evaluated for people with T2D and SMI, which has since been adapted and evaluated in another trial [16]. The small number of trials in the current review that included people with SMI did not specifically address the particular challenges faced by this population, for example, how to maintain diabetes control during periods of instability in their mental health [32, 33]. Furthermore, as the numbers recruited were small, which is not unexpected given the population prevalence of SMI, it is not possible to tell if these interventions were beneficial for people with SMI. These findings together indicate that the provision of evidence-based diabetes education is lacking for people with SMI.

The King’s Fund [34] has recommended that people with SMI should be seen as a priority target group for public health interventions and the UK National Health Service (NHS) Long Term Plan [35] stresses the need for people with SMI to have their physical health needs met. However, our findings concur with other research that has identified under-representation of people with psychiatric disorders in diabetes research and highlighted this issue as an example of health inequity [31]. It is unclear to what extent this under-representation in research on DSME is also borne out in referral to these programmes in clinical care. Research in the US has found that people with SMI are less likely than those without SMI to receive diabetes education [36] but this may not be the case in the UK [33].

Thornicroft [37] has described the mortality gap between people with SMI and the non-SMI population as *“at worst a form of lethal discrimination”* and calls for evidence-based interventions to address it. Furthermore, the Royal College of Psychiatrists [38] has recommended that their members should feel competent to address the physical as well as the mental health needs of people with SMI. Similarly, psychiatric associations internationally [39–41] consider the role of psychiatrists to include improving the physical as well as mental health of their patients. However, the lack of research into the effectiveness of DSME programmes in this vulnerable group leaves clinicians without a clear pathway for intervention.

If people with SMI are to be referred to general DSME programmes, it is essential that the programmes are evaluated in this population. Given the population prevalence of SMI in people with diabetes, recruitment of sufficient people with SMI to achieve adequate statistical power would require trials that oversample people with SMI when recruiting. However, in other research [32, 33] we have identified that learning how to maintain diabetes control during periods of instability in mental health would be an important aspect of DSME for people with SMI. As generic DSME programmes do not address this crucial issue, they will need to be adapted, or more tailored interventions developed and evaluated, if DSME is to meet the needs of people with SMI.

A limitation of this review is that, in spite of our attempts to contact all authors, we were unable to obtain data for a substantial number of trials. It is possible, therefore, that our findings could under- or over-estimate the proportion of trials that do not include SMI. We received responses from authors of 124 RCTs that had not reported data in the trial publication, and of these, 63 (51%) did not have any participants with SMI. If this pattern was repeated for the trials on which we were unable to obtain data, it would raise the proportion who have not included SMI above the 42% reported.

We also acknowledge that we may not have identified all relevant trials, for example, we did not search grey literature or include papers published in languages other than English.

## Conclusion

Very few tailored DSME programmes exist for people with T2DM and SMI therefore people with these conditions may be referred to generic DSME programmes. This systematic review has shown that trials of generic DSME programmes often exclude people with SMI and where they do include people with SMI, efficacy in this population is not tested. We therefore do not know if the DSME programmes to which people with T2DM are referred are effective for people with SMI. If people with SMI are to receive appropriate diabetes care in accordance with current guidelines, it is essential that evidence-based diabetes education is available. It is necessary for future research to examine whether existing programmes can meet the needs of people with SMI, or be adapted to do so, or if more tailored programmes need to be developed and evaluated.

## Supplementary information

**Additional file 1.** Search terms.

**Additional file 2.** Study Characteristics.

## Data Availability

Data are available on request from the corresponding author.

## Abbreviations

A1_c_ or HbA1_c_: Glycosylated haemoglobin
BMI: Body mass index
DSME: Diabetes self-management education
SMI: Severe mental illness
T2DM: Type 2 diabetes mellitus
