# Supplementary material for: Are diabetes self-management programmes for the general diabetes population effective for people with severe mental illness?: a systematic review
Source: BMC Psychiatry. 2020 Jul 25;20:386. doi: 10.1186/s12888-020-02779-7 (PMC7382073; doi:10.1186/s12888-020-02779-7)
Supplement: Supplementary file 1 — Additional file 1. Search terms. [file 12888_2020_2779_MOESM1_ESM.docx]

**Supplementary file 1**

**SEARCH TERMS**

***The Cochrane Library***

# 1MeSH descriptor Diabetes mellitus, type 2 explode all trees

# 2(MODY in All Text or NIDDM in All Text or TDM2 in All Text or TD2 in All Text)

# 3((non in All Text and insulin* in All Text and depend* in All Text) or (noninsulin* in All Text and depend* in All Text) or (non in All Text and insulindepend* in All Text) or noninsulindepend* in All Text)

# 4(typ? in All Text and (2 in All Text near/6 diabet* in All Text))

# 5(typ? in All Text and (II in All Text near/6 diabet* in All Text))

# 6(adult* in All Text near/6 diabet* in All Text)

# 7(matur* in All Text near/6 diabet* in All Text)

# 8(late in All Text near/6 diabet* in All Text)

# 9(slow in All Text near/6 diabet* in All Text)

# 10(stabl* in All Text near/6 diabet* in All Text)

# 11(#1 or #2 or #3 or #4 or #5 or #6 or #7 or #8 or #9 or #10)

# 12MeSH descriptor Diabetes insipidus explode all trees

# 13(diabet* in All Text and insipidus in All Text)

# 14(#12 or #13)

# 15(#11 and not #14)

# 16MeSH descriptor Patient education as topic explode all trees

# 17MeSH descriptor Patient compliance explode all trees

# 18MeSH descriptor Self care explode all trees

# 19MeSH descriptor Health promotion explode all trees

# 20MeSH descriptor Behavior therapy explode all trees

# 21MeSH descriptor Health behavior explode all trees

# 22MeSH descriptor Program evaluation explode all trees

# 23MeSH descriptor Life style explode all trees

# 24((self in All Text near/6 care in All Text) or (self in All Text near/6 monitoring in All Text) or (self in All Text near/6 management in All Text))

# 25((patient in All Text near/6 education* in All Text) or (patient in All Text near/6 compliance in All Text))

# 26((health in All Text near/6 promotion* in All Text) or (health in All Text near/6 behavio* in All Text))

# 27(program in All Text near/6 evaluation* in All Text)

# 28((behavioral in All Text near/6 intervention* in All Text) or (behavioral in All Text near/6 therap* in All Text) or (behavioral in All Text near/6 treatment* in All Text) or (behavioral in Abstract near/6 chang* in Abstract))

# 29((behavioural in All Text near/6 treatment* in All Text) or (behavioural in All Text near/6 therap* in All Text) or (behavioural in All Text near/6 intervention* in All Text) or (behavioural in All Text near/6 chang* in All Text))

# 30((psychosocial in All Text near/6 intervention* in All Text) or (psychosocial in All Text near/6 treatment* in All Text) or (psychosocial in All Text near/6 therap* in All Text))

# 31((psycho-social in All Text near/6 intervention* in All Text) or (psycho-social in All Text near/6 treatment* in All Text) or (psychosocial in All Text near/6 therap* in All Text))

# 32(adherence in All Text or (c in All Text and ompliance in All Text))

# 33((lifestyle in All Text near/6 intervention* in All Text) or (lifestyle in All Text near/6 chang* in All Text))

# 34((life-style in All Text near/6 intervention* in All Text) or (life-style in All Text near/6 chang* in All Text))

# 35MeSH descriptor Weight loss explode all trees

# 36((weight in All Text near/6 management in All Text) or (weight in All Text near/6 los* in All Text) or (weight in All Text near/6 reduction* in All Text))

# 37(#16 or #17 or #18 or #19 or #20 or #21 or #22 or #23 or #24 or #25 or #26 or #27 or #28 or #29 or #30 or #31 or #32 or #33 or #34 or #35 or #36)

# 38(#15 and #37)

**MEDLINE**

1 exp Diabetes Mellitus, Type 2/

2 (MODY or NIDDM or T2DM or T2D).tw,ot.

3 (non insulin$ depend$ or noninsulin$ depend$ or noninsulin?depend$ or non insulin?depend$).tw,ot.

4 ((typ? 2 or typ? II or typ?2 or typ?II) adj3 diabet$).tw,ot.

5 (((late or adult$ or matur$ or slow or stabl$) adj3 onset) and diabet$).tw,ot.

6 or/1-5

7 exp Diabetes Insipidus/

8 diabet$ insipidus.tw,ot.

9 7 or 8

10 6 not 9

11 exp Patient Education as Topic/

12 exp Patient Compliance/

13 exp Self Care/

14 exp Health Promotion/

15 exp Behavior Therapy/

16 exp Health Behavior/

17 exp Program Evaluation/

18 exp Life style/

19 (self adj6 (care or management or monitoring)).tw,ot.

20 (patient adj6 (education* or compliance)).tw,ot.

21 (health adj6 (promotion* or behavio?r*)).tw,ot.

22 (program adj6 evaluation*).tw,ot.

23 (behavio?ral adj6 (intervention* or therap* or treatment* or chang*)).tw,ot.

24 ((psychosocial or psycho-social) adj6 (intervention* or treatment* or therap*)).tw,ot.

25 (adherence or compliance).mp.

26 ((lifestyle or life style) adj6 (intervention* or chang*)).tw,ot.

27 exp Weight Loss/

28 (weight adj6 (management or los* or reduction*)).tw,ot.

29 (lifestyle adj6 (intervention* or chang*)).tw,ot.

30 or/11-29

31 10 and 30

32 randomized controlled trial.pt.

33 controlled clinical trial.pt.

34 randomi?ed.ab.

35 placebo.ab.

36 drug therapy.fs.

37 randomly.ab.

38 trial.ab.

39 groups.ab.

40 or/32-39

41 (comment or editorial or historical-article).pt.

42 40 not 41

43 31 and 42

**EMBASE**

1 exp Diabetes Mellitus, Type 2/

2 (MODY or NIDDM or T2D or T2DM).tw,ot.

3 ((typ? 2 or typ? II or typ?II or typ?2) adj3 diabet*).tw,ot.

4 (obes* adj3 diabet*).tw,ot.

5 (non insulin* depend* or non insulin?depend* or noninsulin* depend* or noninsulin?depend*).tw,ot.

6 ((adult* or matur* or late or slow or stabl*) adj3 diabet*).tw,ot.

7 or/1-6

8 exp diabetes insipidus/

9 diabet* insipidus.tw,ot.

10 8 or 9

11 7 not 10

12 exp patient education/

13 exp patient compliance/

14 exp self care/

15 exp health promotion/

16 exp behavior therapy/

17 *health care quality/

18 exp lifestyle/

19 (self adj6 (care or management or monitoring)).tw,ot.

20 (patient adj6 (education* or compliance)).tw,ot.

21 (health adj6 (promotion* or behavio?r*)).tw,ot.

22 (program adj6 evaluation*).tw,ot.

23 (behavio?ral adj6 (intervention* or therap* or treatment* or chang*)).tw,ot.

24 ((psychosocial or psyco social) adj6 (intervention* or treatment* or therap*)).tw,ot.

25 (adherence or compliance).mp.

26 ((lifestyle or life style) adj6 (intervention* or chang*)).tw,ot.

27 exp weight reduction/

28 (weight adj6 (management or los* or reduction*)).tw,ot.

29 or/12-28

30 11 and 29

31 exp Randomized Controlled Trial/

32 exp Controlled Clinical Trial/

33 exp Clinical Trial/

34 exp Comparative Study/

35 exp Drug comparison/

36 exp Randomization/

37 exp Crossover procedure/

38 exp Double blind procedure/

39 exp Single blind procedure/

40 exp Placebo/

41 exp Prospective Study/

42 ((clinical or control$ or comparativ$ or placebo$ or prospectiv$ or randomi?ed) adj3 (trial$ or stud$)).ab,ti.

43 (random$ adj6 (allocat$ or assign$ or basis or order$)).ab,ti.

44 ((singl$ or doubl$ or trebl$ or tripl$) adj6 (blind$ or mask$)).ab,ti.

45 (cross over or crossover).ab,ti.

46 or/31-45

47 (comment or editorial or historical-article).pt.

48 46 not 47

59 30 and 48

**PsycINFO**

1 exp Diabetes

2 (MODY or NIDDM or T2DM or T2D).tw,ot.

3 (non insulin* depend* or noninsulin* depend* or noninsulin?depend* or non insulin?depend*).tw,ot.

4 ((typ? 2 or typ? II or typ?2 or typ?II) N3 diabet*).tw,ot.

5 (((late or adult* or matur* or slow or stabl*) N3 onset) and diabet*).tw,ot.

6 or/1-5

7 exp Diabetes Insipidus/

8 diabet* insipidus.tw,ot.

9 7 or 8

10 6 not 9

11 exp Self Care/

12 exp Health Promotion/

13 exp Behavior Therapy/

14 exp Health Behavior/

15 exp Program Evaluation/

16 (self N6 (care or management or monitoring)).tw,ot.

17 (patient N6 (education* or compliance)).tw,ot.

18 (health N6 (promotion* or behavio?r*)).tw,ot.

19 (program N6 evaluation*).tw,ot.

20 (behavio?ral N6 (intervention* or therap* or treatment* or chang*)).tw,ot.

21 ((psychosocial or psycho-social) N6 (intervention* or treatment* or therap*)).tw,ot.

22 (adherence or compliance).mp.

23 exp compliance

24 ((lifestyle or life style) N6 (intervention* or chang*)).tw,ot.

25 exp Weight Loss/

26 (weight N6 (management or los* or reduction*)).tw,ot.

27 or/11-26

28 10 and 27

29 randomi?ed.ab.

30 placebo.ab.

31 randomly.ab.

32 trial.ab.

33 groups.ab.

34 or/29-33

35 28 and 34

**AMED**

1. exp diabetes mellitus type 2/

2. (MODY or NIDDM or T2DM or T2D).mp. [mp=abstract, heading words, title]

3. (non insulin$ depend$ or noninsulin$ depend$ or noninsulin?depend$ or non insulin?depend$).mp. [mp=abstract, heading words, title]

4. ((typ? 2 or typ? II or typ?2 or typ?II) adj3 diabet$).mp. [mp=abstract, heading words, title]

5. (((late or adult$ or matur$ or slow or stabl$) adj3 onset) and diabet$).mp. [mp=abstract, heading words, title]

6. 1 or 2 or 3 or 4 or 5

7. exp patient education/

8. exp self care/

9. health education/

10. exp health promotion/

11. exp health behavior/

12. exp program evaluation/

13. exp life style/

14. (self adj6 (care or management or monitoring)).mp. [mp=abstract, heading words, title]

15. (patient adj6 (education* or compliance)).mp. [mp=abstract, heading words, title]

16. (health adj6 (promotion* or behavio?r*)).mp. [mp=abstract, heading words, title]

17. (program adj6 evaluation*).mp. [mp=abstract, heading words, title]

18. (behavio?ral adj6 (intervention* or therap* or treatment* or chang*)).mp. [mp=abstract, heading words, title]

19. ((psychosocial or psycho-social) adj6 (intervention* or treatment* or therap*)).mp. [mp=abstract, heading words, title]

20. (adherence or compliance).mp. [mp=abstract, heading words, title]

21. ((lifestyle or life style) adj6 (intervention* or chang*)).mp. [mp=abstract, heading words, title]

22. exp Weight loss/

23. (weight adj6 (management or los* or reduction*)).mp. [mp=abstract, heading words, title]

24. (lifestyle adj6 (intervention* or chang*)).mp. [mp=abstract, heading words, title]

25. 7 or 8 or 9 or 10 or 11 or 12 or 13 or 14 or 15 or 16 or 17 or 18 or 19 or 20 or 21 or 22 or 23 or 24

26. 6 and 25

27. exp clinical trials/

28. exp comparative study/

29. exp random allocation/

30. exp Placebos/

31. exp prospective studies/

32. ((clinical or control$ or comparativ$ or placebo$ or prospectiv$ or randomi?ed) adj3 (trial$ or stud$)).mp. [mp=abstract, heading

words, title]

33. (random$ adj6 (allocat$ or assign$ or basis or order$)).mp. [mp=abstract, heading words, title]

34. ((singl$ or doubl$ or trebl$ or tripl$) adj6 (blind$ or mask$)).mp. [mp=abstract, heading words, title]

35. (cross over or crossover).mp. [mp=abstract, heading words, title]

36. 27 or 28 or 29 or 30 or 31 or 32 or 33 or 34 or 35

37. 26 and 36

**CINAHL Plus**

S1 (MH “Diabetes Mellitus, Type 2”)

S2 (MODY or NIDDM or T2DM or T2D)

S3 (non insulin* depend* or noninsulin* depend* or noninsulin?depend* or non insulin?depend*)

S4 ((typ* 2 or typ* II or typ#2 or typ#II) N3 diabet*)

S5 (((late or adult* or matur* or slow or stabl*) N3 onset) and diabet*)

S6 S1 or S2 or S3 or S4 or S5

S7 (MH “Patient Education”) OR (MH “Health Education”) OR (MM “Diabetes Education”)

S8 (MH “Self Care”) OR (MH “Self Administration”)

S9 (MH “Health Behavior+”)

S10 (MM “Program Evaluation”)

S11 (MH “Behavior Therapy”)

S12 (MH “Life Style”) OR (MM) “Life Style, Sedentary”) OR (MM “Life Style Changes”)

S13 (MH “Health Promotion”)

S14 (self N6 (care or management or monitoring))

S15 (patient N6 (education* or compliance))

S16 (health N6 (promotion* or behavio#r*))

S17 (program N6 evaluation*)

S18 (behavio#ral N6 (intervention* or therap* or treatment* or chang*))

S19 ((psychosocial or psycho-social) N6 (intervention* or treatment* or therap*))

S20 (adherence or compliance)

S21 ((lifestyle or life style) N6 (intervention* or chang*))

S22 (MH “Weight Loss”)

S23 (MM) “Weight Reduction Programs”)

S24 (weight N6 (management or los* or reduction*))

S25 (lifestyle N6 (intervention* or chang*))

S26 S7 or S8 or S9 or S10 or S11 or S12 or S13 or S14 or S15 or S16 or S17 or S18 or S19 or S20 or S21 or S22 or S23 or S24 or S25

S27 (MH “Clinical Trials+”)

S28 (MM “Comparative Studies”)

S29 (MH “Random Assignment”)

S30 (MH “Prospective Studies”)

S31 ((clinical or control* or comparativ* or placebo* or prospectiv* or randomi#ed) N3 (trial* or stud*))

S32 (random* N6 (allocat* or assign* or basis or order*))

S33 ((singl* or doubl* or trebl* or tripl*) N6 (blind* or mask*))

S34 (cross over or crossover)

S35 S27 or S28 or S29 or S30 or S31 or S32 or S33 or S34

S44 S35 or S43

S45 S6 AND S26 AND S35
