# Supplementary material for: Are diabetes self-management programmes for the general diabetes population effective for people with severe mental illness?: a systematic review
Source: BMC Psychiatry. 2020 Jul 25;20:386. doi: 10.1186/s12888-020-02779-7 (PMC7382073; doi:10.1186/s12888-020-02779-7)
Supplement: Supplementary file 2 — Additional file 2. Study Characteristics. [file 12888_2020_2779_MOESM2_ESM.docx]

STUDY CHARACTERISTICS

|  | Study reference | Country | N start | Trial arms | SMI excluded? | If SMI not excluded, n with SMI |
| --- | --- | --- | --- | --- | --- | --- |
|  | Aas AM, Bergstad I, Thorsby PM, Johannesen Ø, Solberg M, Birkeland KI. Diabetic medicine. 2005 Mar 1;22(3):316-22. | Norway | 38 | 1. Lifestyle intervention  2. Lifestyle intervention plus insulin  3. Insulin alone | NR |  |
|  | Abughosh S., Wang X., Serna O., Esse T et al (2017). Journal of Managed Care & Specialty Pharmacy 23, 5: 549-60 | USA | 5,851 | 1. Motivational interview phone calls 2. Control arm (no motivational interviewing) | NO | NK |
|  | Acik Y, Bulut HY, Gulbayrak C, Ardicoglu O, Ilhan N.. Southeast asian j trop med public health.2004 Dec 35;(4):1012-18 | Turkey | 100 | 1. Diet  2. Diet + exercise  3. Control | NR |  |
|  | Adam, L., O'Connor, C., Garcia, A. (2017). Canadian Journal of Diabetes. | Canada | 21 | 1. Self- management (diabetes conversation map)  2. Traditional education | YES |  |
|  | Adams, K. F., Sperl-Hillen, J. M., Davis, H., Spain, C. V., Hanson, A. M., Fernandes, O. D., ... & Beaton, S. (2013). Diabetes Spectrum, 26(1), 40-45. | USA | 622 | 1. Group education  2. Individual education  3. Control | NR |  |
|  | Adams, S. Y., Crawford, A. G., Rimal, R. N., Lee, J. S., Janneck, L. M., & Sciamanna, C. N. (2009). Population health management, 12(4), 197-204. | USA | 203 | Computer delivery:  1. Efficacy orientated messages  2. Risk orientated messages  3. Delayed treatment control group | NO | NK |
|  | Adepoju, O. E., Bolin, J. N., Phillips, C. D., Zhao, H., Ohsfeldt, R. L., McMaughan, D. K., ... & Forjuoh, S. N. (2014). Patient education and counseling, 95(1), 111-117.  Forjuoh, S., Bolin, J., Huber, J., Vuong, A., Adepoju, O., Helduser, J., Begaye, D et al (2014). BMC Public Health, 14, 71.  Forjuoh, S., Ory, M., Jiang, L., Vuong, A., Bolin, J. (2014). World Journal of Diabetes, 5(3), 407-414 | USA | 376 | 1. CDSMP (Chronic Diabetes Self-management Program)  2. Personal Digital Assistant  3. CDSMP and Personal Digital Assistant  4. Usual care control | NO | NK |
|  | Adepu, R., & Ari, S. M. (2010). Asian J Pharm Clin Res, 3(3), 174-178. | India | 240 | 1. Pharmacist led structured education for diabetes and hypertension  2. Wait list control | NR |  |
|  | Adepu, R., Rasheed, A., & Nagavi, B. G. (2007). Indian Journal of Pharmaceutical Sciences, 69(4), 519. | India | 70 | 1. Pharmacist patient counselling and leaflet  2. Wait list control | NR |  |
|  | Adolfsson, E. T., Walker-Engström, M. L., Smide, B., & Wikblad, K. (2007). Diabetes research and clinical practice, 76(3), 341-350. | Sweden | 137 | 1. Group education 2. Control group 3. Comparison group | NR |  |
|  | Agboola, S., Jethwani, K., Lopez, L., Searl, M., O'Keefe, S., Kvedar, J. (2016). J Med Internet Res, 18 (11):e307 | USA | 126 | 1. Intervention arm (educational text messages)  2. Control arm | YES |  |
|  | Agema, P., & Sherifali, D. (2012). Canadian Journal of Diabetes, 36(4), 199-203. | Canada | 60 | 1. Education and problem solving tool on personal passport 2. Usual care | NO | NK |
|  | Aguiar, P., Chiann, C., Dorea, E., Lyra, D. (2016).  Journal of Evaluation in Clinical Practice, 24(1). | Brazil | 73 | 1. Intervention group (individual, face-to-face pharmaceutical consultations and remote telephone support)  2. Control group | YES |  |
|  | Ahmadi, Z., Sadeghi, T., Loripoor, M. (2018).  Health Education Research, 33(1), 64-72. | Iran | 120 | 1. Peer education group  2. Care provider group  3. Control group | YES |  |
|  | Al-Haj Mohd, M., Phung H., Sun, J., Morisky, D. (2016).  BMC Public health ;16(1):857 16:857 | United Arab Emirates | 446 | 1. Standard care (control group)  2. Educational sessions (intervention group) | NR |  |
|  | Aliha, J. M., Asgari, M., Khayeri, F., Ramazani, M., Farajzadegan, Z., & Javaheri, J. (2013). International journal of preventive medicine, 4(7), 797. | Iran | 62 | 1. Group education and telephone follow-up 2. Usual care | YES |  |
|  | Allen, N. A., Fain, J. A., Braun, B., & Chipkin, S. R. (2008). Diabetes research and clinical practice, 80(3), 371-379. | USA | 52 | 1. Group education and follow-up call PLUS self-efficacy counselling. 2. Group education and follow-up call. | NR |  |
|  | Alotaibi, M., Istepanian, R., Philip, N. (2016). A mobile diabetes management and educational system for type 2 diabetics in Saudi Arabia (SAED). Mhealth, 2(33).  doi: 10.21037/mhealth.2016.08.01 | Saudi Arabia | 20 | 1. Self-management intervention (SAED: diabetes management and education)  2. Control group | NO | NK |
|  | Alvarez, C., Martinez, C., Ramirez-Campillo, R., Rodrigo, M. (2016). International Journal of Sport Medicine, 37(9):723-729 | Chile | 28 | 1. Self-management (HIIT training group)  2. Control group | NR |  |
|  | Amano, Y., Sugiyama, M., Lee, J. S., Kawakubo, K., Mori, K., Tang, A. C., & Akabayashi, A. (2007). Diabetes Care, 30(7), 1874-1876. | Japan | 40 | 1. Group educational session + 4x GI based individual nutrition sessions. 2. Group educational session + 4x conventional nutrition sessions | NR |  |
|  | Amendezo, E., Timothy, W., Karamuka,V., Robinson, B. et al. (2017). Diabetes Research and Clinical Practice, 126, 129-137. | Rwanda | 251 | 1. Self-management (monthly educational sessions)  2. Control group | YES |  |
|  | Arora, S., Peters, A. L., Burner, E., Lam, C. N., & Menchine, M. (2014). Annals of emergency medicine, 63(6), 745-754. | USA | 128 | 1. 2 x daily text messages 2. Usual care | YES |  |
|  | Arslan, M., Ipekci, S., Kebapcilar, L., Dede, N., Kurban, S., Erbay, E., Gonen, M. (2014). International Scholarly Research Notices. Article ID 820387 | Turkey | 64 | 1. Self-management (aerobic exercise group) 2. Control group | YES |  |
|  | Atak N, Gurkan T, Kose K.. Australian Journal of Advanced Nursing, The. 2008 Dec;26(2):66. | Turkey | 80 | 1. Group education 2. Usual care | NR |  |
|  | Avdal, E. U., Kizilci, S., & Demirel, N. (2011). Computers Informatics Nursing, 29(2), 101-106. | Turkey | 122 | 1. Web-based education  2. Control | NR |  |
|  | Ayala, G., Ibarra, L., Cherrington, A. et al. (2015). Outcome of a diabetes control peer support intervention. Ann Fam Med, 1, 9-17. | USA | 336 | 1. Peer support (self-management intervention)  2. Usual care | NR |  |
|  | Aytekin, K., Ovayolu, N., Ovayolu, O. (2016).  Holistic Nursing Practice, 30(2), 70-77. | Turkey | 88 | 1. Self-management intervention (education and follow up telephone call)  2. Control group | YES |  |
|  | Aziz, Z., Riddell, M., Absetz, P., Brand, M., Oldenburg, B. (2018). BMC Public Health, 18, 262 | Australia | 240 | 1. Self-management intervention (peer support for diabetes management)  2. Control group | NR |  |
|  | Babamoto, K. S., Sey, K. A., Camilleri, A. J., Karlan, V. J., Catalasan, J., & Morisky, D. E. (2009). Health Education & Behavior, 36(1), 113-126. | USA | 318 | 1. Community health worker intervention  2. Case management and  3. Standard care | NR |  |
|  | Baghianimoghadam, M. H., Ardekani, M., & Baghianimoghadam, B. (2009). Acta Medica Indonesiana, 41(4), 175-180. | Iran | 120 | 1. Group and individual education 2. Usual care | NR |  |
|  | Baghianimoghadam, M. H., Hadavandkhani, M., Mohammadi, M., Fallahzade, H., & Baghianimoghadam, B. (2012). Rom J Intern Med, 50(2), 165-172. | Iran | 80 | 1. Peer education 2. Group education | NR |  |
|  | Baig, A., Benitez, A., Locklin, C., Gao, Y et al. (2015). J Gen Intern Med, 30(10), 1481-1490. | USA | 100 | 1. 90-minutes diabetes lecture  2. Picture good health (weekly group classes) | YES |  |
|  | Bailey, R., Pfeifer, M., Shillington, A., Harshaw, Q., Funnell, M., VanWingen, J., Col, N. (2016). BMC Health Services Resources, 16, 10. | USA | 225 | 1. Patient decision aid (self-management arm)  2. Usual care | NR |  |
|  | Balducci, S., Zanuso, S., Nicolucci, A., Fernando, F., Cavallo, S., Cardelli, P., ... & Fallucca, F. (2010). Nutrition, Metabolism and Cardiovascular Diseases, 20(8), 608-617. | Italy | 82 | 1. Structured counselling for aerobic low intensity exercise education 2. Exercise group high intensity 3. Exercise group aerobic and resistance 4. Sedentary control | NR |  |
|  | Balducci,S., D'Errico, V., Haxhi, J., Sacchetti, M., Orlando, G. et al. (2017). Diabetes Care, 40(11), 1444-1452. | Italy | 300 | 1. Self-management intervention (theoretical and practical counselling)  2. Control arm | YES |  |
|  | Baradaran, H. R., Knill-Jones, R. P., Wallia, S., & Rodgers, A. (2006) BMC Public Health, 6(1), 134. | UK | 145 | 1. Culturally appropriate (South Asian) group education 2. South Asian usual care control 3. White usual care control | NR |  |
|  | Barceló, A., Cafiero, E., de Boer, M., Mesa, A. E., Lopez, M. G., Jiménez, R. A., ... & Bonfil, G. M. (2010). Primary care diabetes, 4(3), 145-153. | Mexico | 307 | 1. Education groups 2. Usual care | NR |  |
|  | Barratt, R., Frost, G., Millward, D. J., & Truby, H. (2008). British journal of nutrition, 99(05), 1025-1031. | UK | 53 | 1. Dietitian led lifestyle change programme  2. Standard care | NO | NK |
|  | Battista, M. C., Labonté, M., Ménard, J., Jean-Denis, F., Houde, G., Ardilouze, J. L., & Perron, P. (2012). Applied physiology, nutrition, and metabolism, 37(4), 610-620. | Canada | 101 | 1. Additional support and education given by dietitian 2. Usual care | NR |  |
|  | Ben-Avraham, S., Harman-Boehm, I., Schwarzfuchs, D., & Shai, I. (2009). Diabetes research and clinical practice, 86, S41-S48. | Israel | 46 | 1. Mediterranean diet  2. Low fat diet  3. Low carb diet | NR |  |
|  | Bender, M., Cooper, B., Park, L., Padash, S., Arai, S. (2017). JMIR Diabetes, 2(2), e30  DOI: 10.2196/diabetes.8156 | USA | 25 | 1. Mobile-phone based Self-management intervention  2. Control | NR |  |
|  | Beverly EA, Fitzgerald SM, Brooks KM, Hultgren BA, Ganda OP, Munshi M, Weinger K. The Diabetes Educator. 2013 May 2:0145721713486837. | USA | 134 | 1. Group education reinforcing self-care 2. Usual group education | YES |  |
|  | Bieszk, N., Reynolds, S., Wei, W., Davis, C., Kamble, P., Uribe, C. (2016).  Journal of Managed Care & Specialty Pharmacy, 22(9), 1028-1038. | USA | 6243 | 1. Self-management (educational) arm  2. Control group | NR |  |
|  | Bogner, H. R., & de Vries, H. F. (2010). The Diabetes Educator, 36(2), 284-292. | USA | 58 | 1. Integrated care manager providing education about T2D and depression management 2. Usual care | YES |  |
|  | Bogner, H. R., Morales, K. H., de Vries, H. F., & Cappola, A. R. (2012). The Annals of Family Medicine, 10(1), 15-22. | USA | 182 | 1. Integrated care manager providing education about T2D and depression management 2. Usual care | NR |  |
|  | Bollyky, J., Bravata, D., Yang, J., Wiliamson, M., Scheneider, J. (2018). Journal of Diabetes Research, Article ID 3961730 | USA | 330 | 1. Telehealth with no additional support 2. Telehealth plus connected weighing scale 3. Telehealth + weighing scale + lightweight lifestyle coaching 4. Telehealth + weighing scale + intense lifestyle coaching | NR |  |
|  | Bond, G. E., Burr, R., Wolf, F. M., Price, M., McCurry, S. M., & Teri, L. (2007). Diabetes technology & therapeutics, 9(1), 52-59. | USA | 67 | 1. 6-month web-based intervention plus usual care 2. Usual care | NO | NK |
|  | Booth, A., Lowis, C., Hunter, S., Dean, M., Cardwell, C., McKinley, M. (2016). Journal of Diabetes Research, Article ID 3192673 | UK | 84 | 1. Intervention group (lifestyle for diabetes)  2. Control group | NR |  |
|  | Bosi, E., Scavini, M., Ceriello, A., Cucinotta, D, Tiengo, A., Mrino, R. et al. (2013) Diabetes Care, 36, 2887-2894 | Italy | 1024 | 1. Intensive blood sugar monitoring and training 2. Active control | NR |  |
|  | Bowe, M., Cavanaugh, K., Wolff, K., Davis, D., Gregory, R et al (2016).  Patient Education Counselling, 99(8), 1368-1376. | USA | 150 | 1. Control arm 2. Plate Method  3. Carbohydrates counting | YES |  |
|  | Bradshaw, BG, Richardson,GE,l Kumpfer, K., Carlson,J., Stanchfield,J. a Overall, J., Brooks, M and Kulkarni,K (2007) The Diabetes Educator; 33; 650 | USA | 67 | 1. Resiliency education 2. Usual care | YES |  |
|  | Braun, AK, Kubiak, T, Kuntsche, J et al (2009). *Age and Ageing*, 38, 390-396 | Germany | 155 | 1. New treatment and teaching programme 2. Usual care | NR |  |
|  | Brown SA, Blozis, SA et al (2005) *Diabetes care*, 28, 527-532 | USA | 216 | 1. Compressed education  2. Extended education | NO | NK |
|  | Brown, SA., Garcia, A., Winter, M., Silva, L. et al (2011). *Ethnicity & Disease*, 21, 20-26 | USA | 83 | 1. Compressed education 2. Extended education | NO | NK |
|  | Browning, C., Chapman, A., Yang, H., Liu, S. et al. (2016). BMJ Open; 6:e009319. | China | 730 | 1. Telephone and face-to-face motivational interviewing  2. Control group | NR |  |
|  | Buhse, S., Muhlhauser, I., Heller, T., Kuniss, N. et al. (2015). BMJ Open 5(11):e009116 | Germany | 154 | 1. Patient decision aid and education (Self-management of diabetes)  2. Control group | NO | NK |
|  | Bujnowska-Fedak, MM, et al. (2011) *Telemedicine journal and e health*, 17, 153-163 | Poland | 100 | 1. Telehome monitoring and training 2. Usual care | YES |  |
|  | Cade, J.E., Kirk, SFL, Nelson, P., Hollins, L., Deakin, T., Greenwood, DC, et al. (2009) *Diabetic Medicin*e, 26, 1048-1054. | UK | 317 | 1. Expert patient groups  2. Control | NR |  |
|  | Cani, C., Lopes, L., Queiroz, M., Nery, M. (2015). Clinics (Sao Paolo), 70(2), 102-106. | Brazil | 78 | 1. Intervention group (educational program)  2. Control group | NR |  |
|  | Capozza,K., Woolsey, S., Georgsson, M., Black, J., Bello, N., Lence, C., Oostema, S., North, C. (2015). Diabetes Spectrum, 28(2), 83-91. | USA | 93 | 1. Diabetes-related text messages daily  2. Control group | NR |  |
|  | Chai, S., Yao, B., Lin, X., Wang, D., Sun, J., Yuan, N., Zhang, X., Ji, L. (2018).  Patient Education and Counselling, 101, 1427-1432. | China | 118 | 1. Intervention group (educational program) 2. Control group | YES |  |
|  | Chamany, S., Walker, E., Schechter, C., Gonzalez, J et al. (2015). American Journal of Preventative Medicine, 49(6), 832-841. | USA | 933 | 1. Self-management telephone intervention  2. Control (print only) intervention | YES |  |
|  | Chan JC, Sui Y, Oldenburg B, Zhang Y, Chung HH, Goggins W, Au S, Brown N, Ozaki R, Wong RY, Ko GT, Fisher E, JADE and PEARL Project Team. JAMA internal medicine. 2014 Jun 1;174(6):972-81. | China  (Hong Kong) | 628 | 1. Integrated care plus telephone based peer support 2. Integrated care | YES |  |
|  | Chao, J., Yang, L., Xu, H., Yu, Q., Jiang, L., Zong, M. (2015). Archives of Gerontology and Geriatrics, 60, 82-88. | China | 100 | 1. Integrated health management, Self-management group  2. Control group | YES |  |
|  | Chao, YH., Usher, K., Buettner, PG., & Holmes, C (2014) Collegian: Journal of the Royal College of Nursing , Australia, 21, 43-51 | Taiwan | 500 | 1. Booklet plus group education sessions 2. Booklet | NR |  |
|  | Chaveepojnkamjorn W, Pichainarong N, Schelp FP, Mahaweerawat U. Southeast Asian J Trop Med Public Health. 2009 Jan;40(1):169-76. | Thailand | 164 | 1. Group education 2. Usual care | NO | N=0 |
|  | Chee, W., Singh, H., Hamdy, O., Mechanick, J., Lee, V., Barua, A., Ali, S., Hussein, Z. (2017). BMJ Open diabetes research & care. ;5(1):e000384 | Malaysia | 230 | 1. Structured lifestyle intervention with conventional counselling (self-management) 2. Structured lifestyle intervention with motivational interviewing (self-management) 3. Usual Care | NR |  |
|  | Chen, H., Wu, T., Jap, T., Chen, R., & Lin, H. (2008). American Journal of Managed Care, 14(1), 45. | Taiwan | 102 | 1. Individual education 2. usual care and chinese new year pamphlet | NR |  |
|  | Chen, M., Wang, R., Lin, K., Hsu, H., Chen, S. (2015). Applied Nursing Research, 28, 366-373. | Taiwan | 72 | 1. Self-management (empowerment program)  2. Control group | YES |  |
|  | Cheng, L., Sit, J., Choi, K., Chair, S., Li, X., Wu, Y., Long, J., Tao, M. (2018). International Journal of Nursing Studies, 79, 43-51. | China | 242 | 1. Self-management empowerment intervention  2. Control (health education classes) | YES |  |
|  | Chlebowy, D., El-Mallakh, P., Myers, J., Kubiak, N., Cloud, R., Wall, M. (2015). Western Journal of Nursing Research, 37(5), 566-580. | USA | 62 | 1. Self-management (motivational interviewing)  2. Usual care | YES |  |
|  | Cho, J. H., Kwon, H. S., Kim, H. S., Oh, J. A., & Yoon, K. H. (2011). Journal of telemedicine and telecare, 17(7), 365-370. | South Korea | 71 | 1. Remote coaching system via a PDA-type glucometer and the Internet 2. Usual care | YES |  |
|  | Chow, E., Hassali, M., Saleem, F., Aljadhey, H. (2016). Health Education Journal, 75(4), 421-433. | Malaysia | 150 | 1. Self-management (home-based educational intervention)  2. Usual care | YES |  |
|  | Christian, J. G., Bessesen, D. H., Byers, T. E., Christian, K. K., Goldstein, M. G., & Bock, B. C. (2008). Archives of Internal Medicine, 168(2), 141-146. | USA | 155 | 1. Computer assessment/report for patient/physician 2. Usual care | NO | NK |
|  | Cinar, A., Schou, L. (2014). International Dental Journal, 64, 20-28.  Cinar, Ayse Basak; Oktay, Inci; Schou, Lone (2014) Clinical oral investigations, 18 (7) 1793-1801  Cinar, A. B., & Schou, L. (2014). Oral health & preventive dentistry, 12(4). 337-344. | Turkey | 197 | 1. Health coaching  2. Formal oral health education | YES |  |
|  | Clark, M., Hampson, S. E., Avery, L., & Simpson, R. (2004).. British journal of health psychology, 9(3), 365-379. | UK | 100 | 1. Tailored intervention and telephone follow-ups 2. Usual care | YES |  |
|  | Cooper, H., Booth, K., & Gill, G. (2008). Diabetes research and clinical practice, 82(2), 165-171. | UK | 112 | 1. Empowerment based education 2. Wait list control | NR |  |
|  | Coppell KJ, Kataoka M, Williams SM, Chisholm AW, Vorgers SM, Mann JI.. BMJ. 2010 Jul 20;341:c3337. | New Zealand | 104 | 1. Education and enhanced diet 2. Usual care | NR |  |
|  | Cortez, D., Macedo, M., Souza, D., Santos, J., Afonso, G., Reis, I., Torres, H. (2017). BMC Public Health, 17,41.  Santos, J. C. D., Cortez, D. N., Macedo, M. M. L., Reis, E. A., Reis, I. A., & Torres, H. C. (2017). Revista latino-americana de enfermagem, 25. doi: 10.1590/1518-8345.2315.2979 | Brazil | 238 | 1. Empowerment program for self-care, Self-management intervention  2. Control group | YES |  |
|  | Cox, D., Taylor, A., Singh, H., Moncrief, M., Diamond, A., Yancy, W., Hegde, S., McCall, A. (2015). Diabetes Research and Clinical Practice, 111, 28-35. | USA | 39 | 1. Control group  2. Instructional sessions for monitoring blood glucose (Self-management intervention) | YES |  |
|  | Dale, J., Caramlau, I., Sturt, J., Friede, T., & Walker, R. (2009). Patient Education and Counseling, 75(1), 91-98. | UK | 231 | 1. Telecare support provided by peer supporters 2. telecare support provided by diabetes specialist nurses 3. routine care | YES |  |
|  | Dallosso, H., Bodicoat, D., Campbell, M., Carey, M., Davies, M., Eborall, H., Hadjiconstantinou, M., Khunti, K., Speight, J., Heller, S. (2014). Diabetic Medicine, 414-422. | UK | 289 | 1. Self-monitoring of blood glucose  2. Self-monitoring of urine | YES |  |
|  | Daly, M. E., Paisey, R., Millward, B. A., Eccles, C., Williams, K., Hammersley, S., ... & Gale, T. J. (2006). Diabetic Medicine, 23(1), 15-20. | UK | 102 | 1. Group and individual education on low carb diet 2. Group and individual education on healthy eating | NR |  |
|  | Daly, R. M., Dunstan, D. W., Owen, N., Jolley, D., Shaw, J. E., & Zimmet, P. Z. (2005) Osteoporosis international, 16(12), 1703-1712. | Australia | 36 | 1. Supervised high intensity resistance training and weight loss diet 2. Weight loss diet | NO | NK |
|  | Dasgupta, K., Rosenberg, E., Joseph, L., Cooke, A. et al. (2017). Diabetes, Obesity and Metabolism, 19(5), 695-704. | Canada | 347 | 1. Step prescription and pedometer monitoring (Self-management)  2. Control group | NR |  |
|  | Davies, M. J., Heller, S., Skinner, T. C., Campbell, M. J., Carey, M. E., Cradock, S., ... & Khunti, K. (2008)..BMJ, 336(7642), 491-495. | UK | 824 | 1. Structured group education 2. Usual care | YES |  |
|  | De Greef, K., Deforche, B., Tudor-Locke, C., & De Bourdeaudhuij, I. (2010). Health education research, 25(5), 724-736. | Belgium | 62 | 1. Cognitive behavioural programme 2. Usual care | NR |  |
|  | De Greef, K., Deforche, B., Tudor-Locke, C., & De Bourdeaudhuij, I. (2011). International journal of behavioral medicine, 18(3), 188-198. | Belgium | 41 | 1. Individual counselling 2. Group counselling 3. Usual care | YES |  |
|  | De Souza, C., Dalzochio, M., Zucatti, A., Nale, R., Almeida, M., Gross, J., Leitao, C. (2017). Endocrine, 57, 280-286. | Brazil | 118 | 1. Diabetes Education program  2. Control group | NR |  |
|  | Deakin, T. A., Cade, J. E., Williams, R., & Greenwood, D. C. (2006). Diabetic Medicine, 23(9), 944-954. | UK | 314 | 1. Xpert programme delivered to group 2. Control - individual sessions | NO | NK |
|  | Debussche, X., Rollot, O., Le Pommelet, C., Fianu, A., Le Moullec, N., Régnier, C., ... & Favier, F. (2012).Diabetes & metabolism, 38(1), 46-53. | Reunion Island | 398 | 1. Group education 2. Usual care | NO | NK |
|  | Debussche, Xavier; Besancon, Stephane; Balcou-Debussche, Maryvette; Ferdynus, Cyril; Delisle, Helene; Huiart, Laetitia; Sidibe, Assa T. (2018) PloS one, 13 (1) e0191262 | Mali | 151 | 1. Peer structured education 2. Conventional care | YES |  |
|  | Delahanty, Linda M; Dalton, Kristen M; Porneala, Bianca; Chang, Yuchiao; Goldman, Valerie M; Levy, Douglas; Nathan, David M; Wexler, Deborah J. (2015). Obesity (Silver Spring, Md.) 23 (9) 1792-9 | USA | 57 | 1. Diabetes group lifestyle intervention 2. Medical Nutrition Therapy plan | YES |  |
|  | Den Ouden, H., Vos, R., Rutten, G. (2017). Health Expectations, 20(5), 1172-1180. | Netherlands | 153 | 1. Self-management group (goal setting)  2. Control group | YES |  |
|  | Deng, K., Ren, Y., Luo, Z., Du, K., Zhang, X., Zhang, Q. (2016). Medical Science Monitor, 22, 267-275. | China | 208 | 1. Usual care (called traditional group)  2. Self-management (peer support) | YES |  |
|  | Diedrich, A., Munroe, D. J., & Romano, M. (2010). The Diabetes Educator, 36(1), 132-140. | USA | 53 | 1. Pedometer training and group education 2. Group education | NR |  |
|  | Do Rosario Pinto, M., Parreira, P., Basto, M., Monico, L. (2017). BMC Endocrine Disorders, 17, 77. | Portugal | 136 | 1. Educational Self-management program  2. Control group | NR |  |
|  | Dunbar, S., Reilly, C., Gary, R., Higgins, M., Culler, S., Butts, B., Butler, J. (2015).  Journal of Cardiac Failiure, 21(9), 719-729. | USA | 134 | 1. Usual care  2. Educational, Counselling and telephone follow up Self-management intervention | NR |  |
|  | Dutton, G. R., Provost, B. C., Tan, F., & Smith, D. (2008). Preventive medicine, 47(4), 409-411. | USA | 85 | 1. Tailored print based physical activity intervention 2. Usual care | NR |  |
|  | Dyson, P. A., Beatty, S., & Matthews, D. R. (2010). Journal of human nutrition and dietetics, 23(4), 353-359. | UK | 42 | 1. Video education 2. Usual care | YES |  |
|  | Eakin EG, Reeves MM, Winkler E, Healy GN, Dunstan DW, Owen N, Marshal AM, Wilkie KC. Annals of Behavioral Medicine. 2013 Oct 1;46(2):193-203.  Eakin EG, Winkler E, Dunstan DW, Healy GN, Owen N, Marshal AM, Graves N, Reeves MM. Diabetes Care 2014 Aug; 37(8): 2177-2185. | Australia | 302 | 1. Telephone intervention 2. Usual care | YES |  |
|  | Ebrahimi, H., Ashrafi, Z., Rudsari, D., Parsayekta, Z., Haghani, H. (2018).  The Journal of Nursing Research, 26(2), 97-114. | Iran | 80 | 1. Self-management Family-based educational intervention  2. Control group | NR |  |
|  | Ebrahimi, H., Sadeghi, M., Amanpour, F., Vahedi, H. (2016). Primary Care Diabetes, 10, 129-135 | Iran | 106 | 1. Empowerment Self-management  2. Control group | YES |  |
|  | Edelman, D., Dolor, R., Coffman, C., Pereira, K., Granger, B et al (2015).  Journal of General Internal Medicine, 30(5), 626-633. | USA | 377 | 1. Nurse led behavioral self-management intervention  2. Control group | NR |  |
|  | Egede, L., Williams, J., Voronca, D., Gebregziabher, M., Lynch, C. (2017). Journal of General Internal Medicine, 32(7), 775-782. | USA | 255 | 1. Control group  2. Knowledge only group  3. Skills only group  4. Knowledge and Skills group | YES |  |
|  | Erku, D., Ayele, A., Mekuria, A., Belachew, S., Hailemeskel, B., Tegegn, H. (2017). Pharmacy Practice, 15(3), 1026  doi.org/  10.18549/PharmPract.2017.0  3.1026 | Ethiopia | 127 | 1. Pharmacotherapeutic care plan and diabetes education self-management intervention  2. Control group | YES |  |
|  | Essien, O., Otu, A., Umoh, V., Enang, O., Hicks, J., Walley, J. (2017). PLoS ONE 12(1): e0168835. doi:10.1371/journal.  pone.0168835 | Nigeria | 118 | 1. Self-management (intensive education group)  2. Conventional education group | NR |  |
|  | Fan, M., Huang, B., Tang, Y., Han, X., Dong, W., Wang, L. (2016). African Health Sciences, 16(4), 1157-1162. | China | 280 | 1. Self-management (one-to-one education +)  2. Group education in hospital | YES |  |
|  | Fang, W., Rongrui, Z., Yingna, L., Lan, H., Yang, Z., Qing, N., ... & Weidong, W. (2008). Journal of Traditional Chinese Medicine, 28(2), 101-105. | China | 45 | 1. Eight section brocade exercise 2. Usual care | NR |  |
|  | Farahani, M., Purfarzad, Z., Ghorbani, M., Zare, Z., Ghorbani, F. (2016). Clinical Trial. Journal of Caring Sciences, 5(2), 111-120. | Iran | 67 | 1. Diabetes self-care activities and self-care education 2. Control group | NR |  |
|  | Farmer A, Hardeman W, Hughes D, Prevost AT, Kim Y, Craven A, Oke J, Boase S, Selwood M, Kellar I, Graffy J. BMC family practice. 2012 Apr 5;13(1):1. | UK | 211 | 1. Nurse consultation 2. Usual care | NO | NK |
|  | Farmer A, Wade A, Goyder E, Yudkin P, French D, Craven A, Holman R, Kinmonth AL, Neil A. bmj. 2007 Jul 19;335(7611):132. | UK | 453 | 1. Usual care 2. Less intensive self-monitoring 3. Intensive self-monitoring. | NO | NK |
|  | Farsaei S, Sabzghabaee AM, Zargarzadeh AH, Amini M. Journal of Research in Medical Sciences. 2010 Dec 12;16(1):43-9. | Iran | 87 | 1. Educational programme led by pharmacist 2. Usual care | NR |  |
|  | Fayehun, A., Olowookere, O., Ogunbode, A., Adetunji, A., Esan, A. (2018). British Journal of General Practice, 139-147. | Nigeria | 46 | 1. Self-management intervention involving walking prescription  2. Usual care | NO | N=0 |
|  | Fernandes, B., Reis, I., Torres, H. (2016). Revista Latino-Americana De Enfermagem, 24: e2719. | Brazil | 219 | 1. Telephone Self-management for promoting self-care  2. Control group | NR |  |
|  | Fisher L, Polonsky WH, Parkin CG, Jelsovsky Z, Petersen B, Wagner RS. Diabetes research and clinical practice. 2012 May 31;96(2):149-55. | USA | 392 | 1. Structured testing group 2. Active control group | YES |  |
|  | Fisher, L., Hessler, D., Glasgow, R. E., Arean, P. A., Masharani, U., Naranjo, D., & Strycker, L. A. (2013). Diabetes Care, 36(9), 2551-2558. | USA | 583 | 1. Web-based self-management programme 2. web-based, self-management programme plus face to face 3. minimal intervention | YES |  |
|  | Fitzpatrick, S., Golden, S., Stewart, K., Sutherland, J., DeGross, S., Brown, T., Wang, N., Allen, J., Cooper, L., Hill-Briggs, F. (2016). Diabetes Care, 39(12), 2149-2157. | USA | 182 | 1. Decision-making Education for Choices In Diabetes Everyday (DECIDE), education and problem-solving intervention - Self-study  2. DECIDE - Individual  3. DECIDE - group  4. Enhanced Usual Care | NO | N=8 |
|  | Foster GD, Borradaile KE, Vander Veur SS, Shantz KL, Dilks RJ, Goldbacher EM, Oliver TL, LaGrotte CA, Homko C, Satz W. Postgraduate Medicine. 2009 Sep 1;121(5):113-8. | USA | 69 | 1. Portion controlled intervention 2. Group diabetes education | YES |  |
|  | Foster GD, Wadden TA, Lagrotte CA, Vander Veur SS, Hesson LA, Homko CJ, Maschak-Carey BJ, Barbor NR, Bailer B, Diewald L, Komaroff E.. Nutrition & diabetes. 2013 Mar 1;3(3):e63. | USA | 100 | 1. Group lifestyle intervention including portion controlled meal plan 2. Group programme of education | YES |  |
|  | Franciosi M, Lucisano G, Pellegrini F, Cantarello A, Consoli A, Cucco L, Ghidelli R, Sartore G, Sciangula L, Nicolucci A. Diabetic Medicine. 2011 Jul 1;28(7):789-96. | Italy | 62 | 1. Intensive education and self-monitoring of glucose 2. Standard education and no self-monitoring | YES |  |
|  | Frosch DL, Uy V, Ochoa S, Mangione CM. 2011 Dec 12;171(22). | USA | 201 | 1. Intervention included DVD and telephone coaching 2. Educational brochure | NR |  |
|  | Gagliardino JJ, Arrechea V, Assad D, Gagliardino GG, González L, Lucero S, Rizzuti L, Zufriategui Z, Clark C. Diabetes/metabolism research and reviews. 2013 Feb 1;29(2):152-60. | Argentina | 198 | 1. 4-week education course led by peers 2. Same course led by professionals | YES |  |
|  | Gagliardino JJ, Lapertosa S, Pfirter G, Villagra M, Caporale JE, Gonzalez CD, Elgart J, Gonzalez L, Cernadas C, Rucci E, Clark C. Diabetic Medicine. 2013 Sep 1;30(9):1102-11. | Argentina | 468 | 1. Physician education 2. Patient education 3. Both physician and patient education 4. Usual care | YES |  |
|  | Gallagher R, Kirkness A, Zelestis E, Hollams D, Kneale C, Armari E, Bennett T, Daly J, Tofler G. Annals of behavioral medicine. 2012 Aug 1;44(1):119-28. | Australia | 148 | 1. Group education 2. Waiting list control | YES |  |
|  | Gamiochipi, M., Cruz, M., Kumate, J., Wacher, N. (2016). Patient Education and Counselling, 99, 1184-1189. | Mexico | 199 | 1. Intensive Lifestyle intervention  2. Control group (collaborative education model) | YES |  |
|  | Garcia, A., Brown, S., Horner, S., Zuniga, J., Arheart, K. (2015). Health Education Research, 30(3), 484-496. | USA | 72 | 1. Self-management education group  2. Control group | NO | NK |
|  | García-Huidobro D, Bittner M, Brahm P, Puschel K. Family Practice. 2011 Feb 1;28(1):4-11. | Chile | 243 | 1. Families oriented education and counselling 2. Usual care | NO | N=0 |
|  | Garrett N, Hageman CM, Sibley SD, Davern M, Berger M, Brunzell C, Malecha K, Richards SW.. Health Promotion Practice. 2005 Jul 1;6(3):320-8. | USA | 629 | 1. Learning map process group education 2. Self-help manual | NR |  |
|  | Gatwood, J., Balkrishnan, R., Erickson, S., An, L., Piette, J., Farris, K. (2016). Research in Social and Administrative Pharmacy, 12, 130-140. | USA | 75 | 1. Self-management intervention with tailored text-messages  2. Control group | NR |  |
|  | Ghosh S, Rajvanshi A, Kushun S. Int J Pharm Bio Sci. 2010;1:1-6. | India | 22 | 1. Pharmacist counselling intervention 2. Usual care | NR |  |
|  | Gibson, B., Marcus, R. L., Staggers, N., Jones, J., Samore, M., & Weir, C. (2011). Journal of medical Internet research, 14(3), e71-e71. | USA | 65 | 1. Narrated simulation of walking and drawing glucose curve plus intervention of action planning. 2. Control: just narrated simulation | YES |  |
|  | Glasgow RE, Edwards LL, Whitesides H, Carroll N, Sanders TJ, McCray BL. Chronic Illness. 2009 Nov 19. | USA | 189 | 1. DVD education 2. Classroom based education | NO | NK |
|  | Glasgow, R. E., Christiansen, S. M., Kurz, D., King, D. K., Woolley, T., Faber, A. J., ... & Dickman, J. (2011). Journal of medical Internet research, 13(1). | USA | 270 | 1. Website alone 2. Website with human support | NO | NK |
|  | Glasgow, R. E., Kurz, D., King, D., Dickman, J. M., Faber, A. J., Halterman, E., ... & Ritzwoller, D. (2010). Journal of general internal medicine, 25(12), 1315-1322. | USA | 463 | 1. Computer aided 2. Computer aided plus telephone calls etc. 3. Usual care | NO | NK |
|  | Glasgow, R. E., Nutting, P. A., Toobert, D. J., King, D. K., Strycker, L. A., Jex, M., ... & Merenich, J. (2006). Chronic Illness, 2(1), 27-38. | USA | 335 | 1. Tailored self-management computer intervention 2. Computer intervention | NO | NK |
|  | Glasgow, R. E., Strycker, L. A., King, D. K., Toobert, D. J., Rahm, A. K., Jex, M., & Nutting, P. A. (2006).. Am J Manag Care, 12(3), 137-145. | USA | 217 | 1. Computer-assisted behavior change program 2. Enhanced usual care | NO | NK |
|  | Goderis, G., Borgermans, L., Grol, R., Van Den Broeke, C., Boland, B., Verbeke, G., ... & Heyrman, J. (2010). *Diabetes research and clinical practice*, *88*(1), 56-64. | Belgium | 1577 | 1. Patient education 2. Usual care | NR |  |
|  | Goodarzi, M., Ebrahimzadeh, I., Rabi, A., Saedipoor, B., & Jafarabadi, M. A. (2012). Journal of Diabetes & Metabolic Disorders, 11(10). | Iran | 81 | 1. Telephone (SMS) message for health behaviors 2. Usual care | YES |  |
|  | Goode, A. D., Winkler, E. A. H., Lawler, S. P., Reeves, M. M., Owen, N., & Eakin, E. G. (2011). American Journal of Health Promotion, 25(4), 257-263. | Australia | 434 | 1. Telephone calls addressing physical activity and healthy eating 2. Usual care | NR |  |
|  | Goorabi, M., Akhoundan, M., Shadman, Z., Hajifaraji, M., Nikoo, M. (2017). Nutrition and Food Sciences Research, 4(2), 15-23 | Iran | 50 | 1. Self-management nutritional education intervention  2. Control group | NR |  |
|  | Goudswaard, A. N., Stolk, R. P., Zuithoff, N. P. A., De Valk, H. W., & Rutten, G. E. H. M. (2004). Diabetic Medicine, 21(5), 491-496. | Netherlands | 54 | 1. Nurse led education on self-management 2. Usual care | NR |  |
|  | Graumlich, J., Wang, H., Madison, A., Wolf, M., Kaiser, D., Dahal, K., Morrow, D . (2016). Journal of Diabetes Research, Article ID 2129838 | USA | 674 | 1. Self-management intervention group (structured tool that was implemented within the electronic medical record)  2. Usual care | NR |  |
|  | Gregg, J. A., Callaghan, G. M., Hayes, S. C., & Glenn-Lawson, J. L. (2007). Journal of Consulting and Clinical psychology, 75(2), 336. | USA | 81 | 1. Education and Acceptance and Commitment Therapy (ACT) 2. Education alone | NO | N=0 |
|  | Griffiths, C., Motlib, J., Azad, A., Ramsay, J., Eldridge, S., Feder, G., ... & Barlow, J. (2005). British Journal of General Practice, 55(520), 831-837. | UK | 325 | 1. Lay led self-management programme 2. Waiting list control | NR |  |
|  | Grillo, M., Neumann, C., Scain, S., Rozeno, R., Beloli, L., Perinetto, T., Gross, J., Leitao, C. (2016). Diabetes education in primary care: a randomized clinical trial. Cad. Saude Publica, 32(5). | Brazil | 137 | 1.Self-management educational intervention  2. Control group | NR |  |
|  | Guglani, R., Shenoy, S., Sandhu, J. (2014). Journal of Diabetes and Metabolic Disorders, 13, 110. | India | 102 | 1. Supervised exercise pedometer group  2.Self-reported exercise group with pedometer  3. Control group | YES |  |
|  | Guo, H., Tian, X., Li, R., Lin, J., Jin, N., Wu, Z., Yu, D. (2014). Journal of Diabetes Investigation, 5(4), 410-417. | China | 132 | 1. Glucose self-monitoring group at no cost  2. Glucose self-monitoring at conditional cost  3. Control group | YES |  |
|  | Guo, X. H., Ji, L. N., Lu, J. M., Liu, J., Lou, Q. Q., Liu, J., ... & Gu, M. J. (2014). Journal of diabetes, 6(4), 290-297. | China | 1511 | 1. Education 2. Usual care | YES |  |
|  | Gutschall, M. D., Miller, C. K., Mitchell, D. C., & Lawrence, F. R. (2009). Public health nutrition, 12(10), 1846-1854. | USA | 109 | 1. Group education 2. Waiting list control | NR |  |
|  | Habibzadeh, H., Sofiani, A., Alilu, L., Gillespie, M. (2017). Oman Medical Journal, 32(6), 499-506. | Iran | 90 | 1. Self-management intervention group (group-discussion based education)  2. Usual care | YES |  |
|  | Hamid, S., Dunsiger, S., Seiden, A., Nu'usolia, O., Tuitele, J., DePue, J. D., & McGarvey, S. T. (2013). Chronic illness, 1742395313502367. | American Samoa | 268 | 1. Nurse-community health worker (CHW) intervention 2. Usual care | NO | NK |
|  | Handley, M. A., Shumway, M., & Schillinger, D. (2008). The Annals of Family Medicine, 6(6), 512-518. | USA | 226 | 1. Automated telephone self-management support plus nurse care management 2. Usual care | YES |  |
|  | Hansen, C., Perrlld, H., Koefoed, B., Zander, M. (2017). Clinical and Translational Endocrinology, 176(6), 727-736. | Denmark | 165 | 1. Telemedicine self-management group  2. Control group | NO | NK |
|  | Hare JL, Hordern MD, Leano R, Stanton T, Prins JB, Marwick TH. 2011 May 16:CIRCHEARTFAILURE-110. | Australia | 223 | 1. Supervised exercise program and lifestyle 2. usual care | NR |  |
|  | Heinrich, E., de Nooijer, J., Schaper, N. C., Schoonus-Spit, M. H., Janssen, M. A., & de Vries, N. K. (2012). Patient education and counseling, 86(2), 172-178. | Netherlands | 135 | 1. Web site education and self-management evaluated by online questionnaire 2. Control group 3. Post-test only control group | NR |  |
|  | Hermanns, N., Ehrmann, D., Schall, S., Maier, B., Haak, T., Kulzer, B. (2017). Diabetic Medicine, 34, 1084-1091. | Germany | 182 | 1. Control group  2. Self-management education intervention | YES |  |
|  | Hermanns, N., Kulzer, B., Maier, B., Mahr, M., & Haak, T. (2012). Patient education and counseling, 86(2), 226-232. | Germany | 186 | 1. Novel education programme 2. Established education programme | YES |  |
|  | Hill-Briggs, F., Lazo, M., Peyrot, M., Doswell, A., Chang, Y. T., Hill, M. N., ... & Brancati, F. L. (2011). Journal of general internal medicine, 26(9), 972-978. | USA | 56 | 1. Decision-making Education for Choices In Diabetes Everyday (DECIDE) - Intensive education and problem-solving intervention 2. DECIDE - Condensed intervention | NO | N=4 |
|  | Hörnsten, Å., Stenlund, H., Lundman, B., & Sandström, H. (2008). Diabetes research and clinical practice, 81(1), 50-55. | Sweden | 104 | 1. Group education 2. Usual care | YES |  |
|  | Hosseini, S. G., Shojaeizadeh, D., Sanagu, A., Vakili, M. A., Mirkarimi, K., & Jahanshahi, R. (2017). Annals of Tropical Medicine and Public Health, 10(3), 707. | Iran | 106 | 1. Educational intervention on self-care behaviours  2. Routine education | NR |  |
|  | House, A., Bryant, L., Russell, A. M., Wright‐Hughes, A., Graham, L., Walwyn, R., ... & Stansfield, A. (2018). Diabetic Medicine, 35(6), 776-788. doi: 10.1111/dme.13626 | UK | 82 | 1. Intervention group- supported self-management  2. Control group- Usual care | NO | NK |
|  | Hsu, C. C., & Tai, T. Y. (2014). Diabetes research and clinical practice, 106, S328-S332. | Taiwan | 1060 | 1. Intervention group- self-care and nutrition-education  2. Control group- standard care group | NO | NK |
|  | Huang, M. C., Hsu, C. C., Wang, H. S., & Shin, S. J. (2010). Diabetes care, 33(2), 233-239. | Taiwan | 154 | 1. Nutrition education 2. Routine care | NR |  |
|  | Huisman, S., Maes, S., De Gucht, V. J., Chatrou, M., & Haak, H. R. (2010). International journal of behavioral medicine, 17(3), 176-181. | Netherlands | 88 | 1. Self-regulation intervention 2. active control group 3. passive control group. | YES |  |
|  | Huizinga, M. M., Gebretsadik, T., Ulen, C. G., Shintani, A. K., Michon, S. R., Shackleford, L. O., ... & Elasy, T. A. (2010). Diabetologia, 53(5), 832-839. | USA | 165 | 1. Routine follow up and quarterly telephone call 2. Routine follow up and monthly telephone call 3. Routine follow up | NR |  |
|  | Iljaž, R., Brodnik, A., Zrimec, T., & Cukjati, I. (2017). Slovenian Journal of Public Health, 56(3), 150–157. doi: 10.1515/sjph-2017-0020 | Slovenia | 120 | 1. eDiabetes – telemonitoring, e-consultation and education  2. Usual care | NO | N=6 |
|  | Istepanian, R.S., Sitouni, K., Harry, D., Moutosammy, N., Sungour, A., Tang, B. et al. (2009). Journal of Telemedicine & Telecare, 15, 125-128. | UK | 137 | 1. Telemonitoring 2. Usual care | NR |  |
|  | Izquierdo, R., Lagua, C. T., Meyer, S., Ploutz-Snyder, R. J., Palmas, W., Eimicke, J. P., ... & Weinstock, R. S. (2010). Diabetes technology & therapeutics, 12(3), 213-220. | USA | 890 | 1. Home televisits 2. Usual care | NR |  |
|  | Jaipakdee, J., Jiamjarasrangsi, W., Lohsoonthorn, V., & Lertmaharit, S. (2015). Nursing & health sciences, 17(3), 362-369.  doi: 10.1111/nhs.12198 | Thailand | 403 | 1. Intervention group- diabetes self-management support  2. Control group- Usual care | YES |  |
|  | Jansink, R., Braspenning, J., Keizer, E., van der Weijden, T., Elwyn, G., & Grol, R. (2013). Scandinavian journal of primary health care, 31(2), 119-127. | Netherlands | 521 | 1. Intensive lifestyle intervention 2. Diabetes support and education | YES |  |
|  | Jayasuriya, R., Pinidiyapathirage, M. J., Jayawardena, R., Kasturiratne, A., de Zoysa, P., Godamunne, P., ... & Wickremasinghe, A. R. (2015). Primary care diabetes, 9(5), 338-345. doi: 10.1016/j.pcd.2015.01.014 | Sri Lanka | 85 | 1. Diabetes Self-Management-Sri Lanka (DSM-SL)  2. Usual care | YES |  |
|  | Jennings, C. A., Vandelanotte, C., Caperchione, C. M., & Mummery, W. K. (2014). 60: 33-40. | Australia | 397 | 1. Fully automated web-based programme to increase physical activity 2. Modified version of the website | NO | NK |
|  | Jiang, X., Fan, X., Wu, R., Geng, F., & Hu, C. (2017). The effect of care intervention for obese patients with type II diabetes. Medicine, 96(42), e7524. doi: 10.1097/MD.0000000000007524 | China | 126 | 1. Dietary, exercise, and psychology interventions  2. Conventional care | YES |  |
|  | Johansen, M. Y., MacDonald, C. S., Hansen, K. B., Karstoft, K., Christensen, R., Pedersen, M., … Ried-Larsen, M. (2017). JAMA, 318(7), 637–646. doi: 10.1001/jama.2017.10169 | Denmark | 98 | 1. Intervention group- standard care plus 5-6 weekly aerobic sessions, a dietary plan, dietary counseling  2. Control group- standard care | YES |  |
|  | Johansen, O. E., Gullestad, L., Blaasaas, K. G., Orvik, E., & Birkeland, K. I. (2007). Diabetic Medicine, 24(9), 1019-1027. | Norway | 120 | 1. Structured care for life style modification 2. Usual care | NR |  |
|  | Jutterström, L., Hörnsten, Å., Sandström, H., Stenlund, H., & Isaksson, U. (2016). Patient Education and Counseling, 99(11), 1821-1829. doi: 10.1016/j.pec.2016.06.016 | Sweden | 572 | 1.Group self-management intervention  2. Individual self-management intervention  3. Standard care internal control group  4. External control group- recruited from another county council | YES |  |
|  | Kadoglou, N. P., Vrabas, I. S., Kapelouzou, A., Lampropoulos, S., Sailer, N., Kostakis, A., ... & Angelopoulou, N. (2012). Medical Science Monitor Basic Research, 18(5), CR290-CR295. | Greece | 53 | 1. Structured exercise training 4 times a week 2. Verbal instruction | NR |  |
|  | Karhula, T., Vuorinen, A.-L., Rääpysjärvi, K., Pakanen, M., Itkonen, P., Tepponen, M., … Saranummi, N. (2015). Journal of Medical Internet Research, 17(6), e153. doi: 10.2196/jmir.4059 | Finland | 287 | 1. Health coaching over the phone and self-monitoring of health parameters using remote patient monitoring (RPM)  2. Usual care | NO | NK |
|  | Keeratiyutawong, P., Hanucharurnkul, S., Melkus, G. D. E., Panpakdee, O., & Vorapongsathorn, T. (2006). Thai J Nurs Res, 10(2), 85-97. | Thailand | 90 | 1. Self-management programme 2. Usual care | NR |  |
|  | Kempf, K., Altpeter, B., Berger, J., Reuß, O., Fuchs, M., Schneider, M., ... & Martin, S. (2017). Diabetes care, 40(7), 863-871. doi: 10.2337/dc17-0303 | Germany | 202 | 1. Intervention group- Telemedical Lifestyle intervention Program (telipro  2. Weighing scales, step counters and remained in routine care | YES |  |
|  | Keogh, K. M., Smith, S. M., White, P., McGilloway, S., Kelly, A., Gibney, J., & O'Dowd, T. (2011). The American journal of managed care, 17(2), 105-113. | Ireland | 121 | 1. Family based motivational interviewing educational intervention 2. Usual care | NR |  |
|  | Kim, M. T., Han, H. R., Song, H. J., Lee, J. E., Kim, J., Ryu, J. P., & Kim, K. B. (2009). The Diabetes Educator, 35(6), 986-994. | USA | 83 | 1. 6-week education programme self-monitoring, and follow-up counselling 2. Waiting list intervention | NR |  |
|  | Kim, M. T., Kim, K. B., Huh, B., Nguyen, T., Han, H.-R., Bone, L. R., & Levine, D. (2015). American Journal of Preventive Medicine, 49(5), 726–737. doi: 10.1016/j.amepre.2015.04.033 | USA | 250 | 1. Community-based, multimodal behavioral self-help intervention program  2. Waitlist control | NO | NK |
|  | Kim, S. H., Lee, S. H., Ahn, K. Y., Lee, D. H., Suh, Y. J., Cho, S. G., ... & Kim, Y. S. (2014). Clinical endocrinology, 80(6), 825-833. | South Korea | 35 | 1. Intensive lifestyle modiﬁcation 2. Usual care | NO | N=0 |
|  | Kim, S. H., Lee, S. J., Kang, E. S., Kang, S., Hur, K. Y., Lee, H. J., ... & Lee, H. C. (2006). Metabolism, 55(8), 1053-1059. | South Korea | 58 | 1. Diet, exercise, and behavior modification 2. Usual care and 1 educational session | YES |  |
|  | Kim, S. I., & Kim, H. S. (2008). International Journal of medical informatics, 77(6), 399-404. | South Korea | 34 | 1. SMS messages reporting on glucose, and education 2. Usual care | NR |  |
|  | King, A. B., Wolfe, G. S., & Armstrong, D. U. (2007). Diabetes technology & therapeutics, 9(3), 241-245. | USA | 30 | 1. Simple start DVD and booklet and education 2. Education alone | YES |  |
|  | Kirk, A. F., Mutrie, N., MacIntyre, P. D., & Fisher, M. B. (2004). American Journal of Preventive Medicine, 27(4), 289-296. | UK | 70 | 1. Exercise consultation and standard leaflet 2. Standard leaflet only | NO | N=0 |
|  | Kirk, A., Barnett, J., Leese, G., & Mutrie, N. (2009). Diabetic Medicine, 26(3), 293-301. | UK | 134 | 1. Physical Activity Consultation intervention given face to face 2. Or written 3. Usual care | NO | N=0 |
|  | Kirk, J. K., Craven, T., Lipkin, E. W., Katula, J., Pedley, C., O’Connor, P. J., & Margolis, K. L. (2013). Diabetes research and clinical practice, 100(1), 61-68. | USA | 1781 | 1. Dietary counselling for low fat diet intensive treatment group 2. Standard treatment group | NR |  |
|  | Ko, G. T. C., Li, J. K. Y., Kan, E. C. Y., & Lo, M. K. W. (2004). Diabetic medicine, 21(12), 1274-1279. | China  (Hong Kong) | 178 | 1. Structured health education programme by a trained diabetic education nurse 2. Usual care | NR |  |
|  | Ko, S. H., Song, K. H., Kim, S. R., Lee, J. M., Kim, J. S., Shin, J. H., ... & Cha, B. Y. (2007). Diabetic medicine, 24(1), 55-62. | South Korea | 437 | 1. Intensive inpatient education for self-management 2. Usual care | YES |  |
|  | Koekkoek, P. S., Ruis, C., van den Donk, M., Biessels, G. J., Gorter, K. J., Kappelle, L. J., & Rutten, G. E. (2012). Journal of the neurological sciences, 314(1), 71-77. | Netherlands | 498 | 1. Intensive multifactorial treatment 2. Usual care | YES |  |
|  | Korcegez, E. I., Sancar, M., & Demirkan, K. (2017). Journal of managed care & specialty pharmacy, 23(5), 573-582. doi: 10.18553/jmcp.2017.23.5.573 | Cyprus | 159 | 1. Pharmacist-led program  2. Usual care | YES |  |
|  | Krakow, D., & Feulner‐Krakow, G. (2007). European Diabetes Nursing, 4(3), 106-112. | Germany | 823 | 1. New education programme LINDA 2. Usual care | YES |  |
|  | Krebs, J. D., Elley, C. R., Parry-Strong, A., Lunt, H., Drury, P. L., Bell, D. A., ... & Mann, J. I. (2012). Diabetologia, 55(4), 905-914. | New Zealand | 419 | 1. High carbohydrate diet education 2. High protein diet education | YES |  |
|  | Kroese, F. M., Adriaanse, M. A., & De Ridder, D. T. (2013). Health Education & Behavior, 40(5), 552-558. | Netherlands | 64 | 1. Education programme with obese patients 2. Education programme with non obese patients | YES |  |
|  | Kulzer, B., Hermanns, N., Reinecker, H., & Haak, T. (2007). Diabetic Medicine, 24(4), 415-423. | Germany | 193 | 1. Didactic training programme 2. Self-management training programme 3. Individualised approach | YES |  |
|  | Kuznetsov, L., Simmons, R.K., Sutton, S., Kinmonth, A.L., Griffin, S.J. and Hardeman, W., 2013. International Journal of Behavioral Nutrition and Physical Activity, 10(1), p.1.  Griffin, S., Simmons, R., Prevost, T., Williams, K., Hardeman, W. et al (2014). Diabetology, 57(7), 1308-1319. | UK | 478 | 1. Intensive treatment plus behaviour change intervention 2. Intensive treatment | YES |  |
|  | Labrunée M, Antoine D, Verges B, Robin I, Casillas JM, Gremeaux V. Annals of physical and rehabilitation medicine. 2012 Sep 30;55(6):415-29. | France | 23 | 1. Tailored program of physical training 2. Usual care | NR |  |
|  | Lamers, F., Jonkers, C., Bosma, H., Knottnerus, J. A., & van Eijk, J. T. M. (2011). Journal of advanced nursing, 67(4), 788-799. | Netherlands | 208 | 1. Nurse administered minimal psychological intervention 2. Care as usual | YES |  |
|  | Larsen, R. N., Mann, N. J., Maclean, E., & Shaw, J. E. (2011). Diabetologia, 54(4), 731-740. | Australia | 99 | 1. Low fat high protein diet education 2. Low fat high carbohydrate diet education | NR |  |
|  | Lawler, S. P., Winkler, E. A., Goode, A. D., Fjeldsoe, B. S., Reeves, M. M., & Eakin, E. G. (2014). Preventive medicine, 61, 34-41. | Australia | 417 | 1. Manual, pedometer, telephone support 2. Usual care plus written info | YES |  |
|  | Lean, M. E., Leslie, W. S., Barnes, A. C., Brosnahan, N., Thom, G., McCombie, L., ... & Taylor, R.. (2018). Lancet, 391(10120), 541-551. doi: 10.1016/ S0140-6736(17)33102-1 | UK | 306 | 1. Weight management programme  2. Best-practice care by guidelines | NR |  |
|  | LeMaster, J. W., Mueller, M. J., Reiber, G. E., Mehr, D. R., Madsen, R. W., & Conn, V. S. (2008). Physical Therapy, 88(11), 1385-1398. | USA | 79 | 1. Physio and nurse visits, motivational phone calls. Graded walking exercise. 2. Foot care advice | NR |  |
|  | Leu, M. G., Norris, T. E., Hummel, J., Isaac, M., & Brogan, M. W. (2005). Diabetes technology & therapeutics, 7(5), 710-718. | USA | 50 | 1. Pager messages as requested by patient 2. Usual care | NR |  |
|  | Li, M., Li, T., Shi, B. Y., & Gao, C. X. (2014). International journal of nursing sciences, 1(3), 250-254. doi: 10.1016/j.ijnss.2014.05.022 | China | 101 | 1. Motivational interviewing  2. Routine diabetes education | NR |  |
|  | Liang, R., Dai, X., Zuojie, L., Zhou, A., & Meijuan, C. (2012). Canadian Journal of Diabetes, 36(1), 15-18. | China | 62 | 1. Foot care kit and self-management education 2. Usual care | NR |  |
|  | Lincoln, N. B., Radford, K. A., Game, F. L., & Jeffcoate, W. J. (2008). Diabetologia, 51(11), 1954-1961. | UK | 172 | 1. Foot care visit and follow up phone call. Illustrated handouts. 2. Usual care | NR |  |
|  | Liu, S., Bi, A., Fu, D., Fu, H., Luo, W., Ma, X., & Zhuang, L. (2012). BMC public health, 12(1), 1043. | China | 208 | 1. Education for self-management 2. Usual care | YES |  |
|  | Logtenberg, S. J., Kleefstra, N., Houweling, S. T., Groenier, K. H., & Bilo, H. J. (2007). Journal of hypertension, 25(1), 241-246. | Netherlands | 30 | 1. Device-guided breathing exercises 2. Music | NO | N=0 |
|  | Lorig, K., Ritter, P. L., Laurent, D. D., Plant, K., Green, M., Jernigan, V. B. B., & Case, S. (2010). Diabetes care, 33(6), 1275-1281. | USA | 110 | 1. Online diabetes self-management program | NR |  |
|  | Lorig, K., Ritter, P. L., Villa, F. J., & Armas, J. (2009). The Diabetes Educator, 35(4), 641-651. | USA | 345 | 1. Peer led educational self-management education 2. Usual care | NR |  |
|  | Lorig, K., Ritter, P. L., Villa, F., & Piette, J. D. (2008). Diabetes Care, 31(3), 408-414. | USA | 417 | 1. Tailored Spanish Diabetes self-management (SDSM)education programme 2. Usual care 3. Telephone reinforcement of the SDSMP 4. Non-reinforced SDSMP | NR |  |
|  | Lu, J., Bu, R. F., Sun, Z. L., Lu, Q. S., Jin, H., Wang, Y., ... & Yang, B. Q. (2011). Diabetes research and clinical practice, 93(2), 179-186. | China | 108 | 1. Self-care education enhanced (urine glucose monitoring) 2. Self-care education (blood glucose monitoring) 3. Control – no monitoring | YES |  |
|  | Luley, C., Blaik, A., Reschke, K., Klose, S., & Westphal, S. (2011). Diabetes research and clinical practice, 91(3), 286-292. | Germany | 68 | 1. Group education 2. Usual care | YES |  |
|  | Lutes, L. D., Cummings, D. M., Littlewood, K., Dinatale, E., & Hambidge, B. (2017). Obesity, 25(8), 1329-1335. | USA | 200 | 1. Lifestyle intervention sessions aimed at making small changes in their diet and activity  2. Educational mailings | YES |  |
|  | Lyons, I., Barber, N., Raynor, D. K., & Wei, L. (2016). BMJ Qual Saf, 25, 759–769. doi:10.1136/bmjqs-2015-004670 | UK | 677 | 1. Pharmacist-led telephone based intervention  2. Standard care | NR |  |
|  | Ma, Y., Olendzki, B. C., Merriam, P. A., Chiriboga, D. E., Culver, A. L., Li, W., ... & Pagoto, S. L. (2008). Nutrition, 24(1), 45-56. | USA | 40 | 1. Low-GI diet education 2. Standard ADA diet education | NR |  |
|  | Macedo, M. M. L., Cortez, D. N., Santos, J. C. D., Reis, I. A., & Torres, H. D. C. (2017). Revista da Escola de Enfermagem da USP, 51. doi:10.1590/S1980-220X2016050303278 | Brazil | 200 | 1. Seven group meetings  2. Usual care | NR |  |
|  | Madden, K. M., Lockhart, C., Cuff, D., Potter, T. F., & Meneilly, G. S. (2009). Diabetes Care, 32(8), 1531-1535. | Canada | 52 | 1. Aerobic exercise training 2. Non aerobic exercise | NO | N=0 |
|  | Mahdizadeh, M. S., Peymam, N., Taghipour, A., Esmaily, H., & Mahdizadeh, S. M. (2013). Journal of research in health sciences, 13(1), 90-97. | Iran | 82 | 1. Intensive education 2. Control – written information | YES |  |
|  | Malanda, U. L., Bot, S. D. M., Kostense, P. J., Snoek, F. J., Dekker, J. M., & Nijpels, G. (2016). Diabetic Medicine, 33(4), 537-546. | Netherlands | 181 | 1. Blood monitoring group  2. Urine monitoring group  3. Usual care | YES |  |
|  | Malathy, R., Narmadha, M. P., Jose, M. A., Ramesh, S., & Babu, N. D. (2011). Journal of Young pharmacists, 3(1), 65-72. | India | 207 | 1. Pharmacist counselling sessions 2. Waiting list control | NR |  |
|  | Maljanian, R., Grey, N., Staff, I., & Conroy, L. (2005). Disease Management, 8(1), 15-25. | USA | 336 | 1. Telephone support for lifestyle intervention 2. Lifestyle intervention | NR |  |
|  | Markle‐Reid, M., Ploeg, J., Fraser, K. D., Fisher, K. A., Bartholomew, A., Griffith, L. E., … Upshur, R. (2018). Journal of the American Geriatrics Society, 66(2), 263–273. doi: 10.1111/jgs.15173 | Canada | 159 | 1. Community Program  2. Usual care | NO | NK |
|  | Mash, R. J., Rhode, H., Zwarenstein, M., Rollnick, S., Lombard, C., Steyn, K., & Levitt, N. (2014). Diabetic Medicine, 31(8), 987-993. doi: 10.1111/dme.12475 | South Africa | 1570 | 1. Group diabetes education led by a health promoter  2. Usual care | YES |  |
|  | Maslakpak, M., Razmara, S., Niazkhani, Z. (2017). Journal of Diabetes Research, 10 pages. | Iran | 90 | 1. Face to Face education group  2. Phone-based Education group  3. Control group | YES |  |
|  | Mayer-Davis, E. J., D'Antonio, A. M., Smith, S. M., Kirkner, G., Levin Martin, S., Parra-Medina, D., & Schultz, R. (2004). American Journal of Public Health, 94(10), 1736-1742. | USA | 152 | 1. Intensive lifestyle intervention 2. Condensed lifestyle intervention | YES |  |
|  | McGowan, P. (2011). Canadian Journal of Diabetes, 35(1), 46-53. | Canada | 234 | 1. Didactic model of diabetes patient education provided at a diabetes education 2. Diabetes patient education with a community self-management program | NR |  |
|  | McMahon, G. T., Fonda, S. J., Gomes, H. E., Alexis, G., & Conlin, P. R. (2012). Diabetes technology & therapeutics, 14(11), 1060-1067. | USA | 152 | 1. Telephone or online care management 2. Usual care supplemented with internet access and training. | NR |  |
|  | Mehuys, E., Van Bortel, L., De Bolle, L., Van Tongelen, I., Annemans, L., Remon, J. P., & Giri, M. (2011). Journal of clinical pharmacy and therapeutics, 36(5), 602-613. | Belgium | 288 | 1. Education by pharmacist 2. Usual care | NO | NK |
|  | Ménard, J., Payette, H., Baillargeon, J. P., Maheux, P., Lepage, S., Tessier, D., & Ardilouze, J. L. (2005). Canadian Medical Association Journal, 173(12), 1457-1466. | Canada | 72 | 1. Multidisciplinary team intervention for lifestyle education 2. Usual care | YES |  |
|  | Miller, C. K., Kristeller, J. L., Headings, A., Nagaraja, H., & Miser, W. F. (2012). Journal of the Academy of Nutrition and Dietetics, 112(11), 1835-1842.  Miller, C. K., Kristeller, J. L., Headings, A., Nagaraja, H (2014). Health Educ Behav. 2014 Apr; 41(2): 145–154. | USA | 52 | 1. Mindful eating intervention 2. Diabetes self-management education | YES |  |
|  | Mohamed, H., Al-Lenjawi, B., Amuna, P., Zotor, F., & Elmahdi, H. (2013). Primary care diabetes, 7(3), 199-206. | Qatar | 430 | 1. Culturally sensitive, structured education programme 2. Control group - Educational tool kit | NO | N=5 |
|  | Mollaoğlu, M., & Beyazıt, E. (2009). Applied nursing research, 22(3), 183-190. | Turkey | 50 | 1. Nurse-initiated planned education 2. Usual care | NR |  |
|  | Monteiro, L. Z., Fiani, C. R. V., Freitas, M. C. F. D., Zanetti, M. L., & Foss, M. C. (2010). Arquivos brasileiros de cardiologia, 95(5), 563-570. | Brazil | 22 | 1. Aerobic training 2. Educational lectures | NR |  |
|  | Moriyama, M., Nakano, M., Kuroe, Y., Nin, K., Niitani, M., & Nakaya, T. (2009). Japan Journal of Nursing Science, 6(1), 51-63. | Japan | 65 | 1. Manual and face to face contact 2. Control - manual | YES |  |
|  | Muller, I., The Diabetes Literacy Study Group, I., Rowsell, A., Stuart, B., Hayter, V., Little, P., Ganahl, K., … Yardley, L. (2017). Journal of Medical Internet Research, 19(1), e21. doi: 10.2196/jmir.6601 | UK, Austria, Germany, Ireland, and Taiwan | 1045 | 1. Intervention group- Interactive intervention  2. Control group- Static intervention | YES |  |
|  | Munshi, M. N., Segal, A. R., Suhl, E., Ryan, C., Sternthal, A., Giusti, J., ... & DesRochers, L. (2013) Diabetes care, 36(3), 543-549. | USA | 100 | 1. Telephone calls assessing barriers 2. Home visits assessing barriers 3. Attention control | NR |  |
|  | Murray, E., Sweeting, M., Dack, C., Pal, K., Modrow, K., Hudda, M., ... & Farmer, A. (2017). BMJ open, 7(9), e016009. doi: 10.1136/bmjopen-2017- 016009 | UK | 374 | 1. Intervention group- Healthy Living for People with Diabetes (help- Diabetes)  2. Control group- regular care | NO | NK |
|  | Nagrebetsky, A., Larsen, M., Craven, A., Turner, J., McRobert, N., Murray, E., ... & Farmer, A. (2013). Journal of diabetes science and technology, 7(1), 123-134. | UK | 17 | 1. Education and training in glucose monitoring by telehealth 2. Education | NO | NK |
|  | Naik, A. D., Teal, C. R., Rodriguez, E., & Haidet, P. (2011). Patient education and counseling, 85(3), 383-389.  Naik, A. D., Palmer, N., Petersen, N. J., Street, R. L., Rao, R., Suarez-Almazor, M., & Haidet, P. (2011). Archives of internal medicine, 171(5), 453-459. | USA | 87 | 1. Empowerment intervention 2. Traditional diabetes education | NR |  |
|  | Nam, S., Dobrosielski, D. A., & Stewart, K. J. (2012). Journal of cardiopulmonary rehabilitation and prevention, 32(6), 370. | USA | 140 | 1. Written info on exercise and training 3 x a week 2. Only written info | NR |  |
|  | Nebel, I. T., Klemm, T., Fasshauer, M., Müller, U., Verlohren, H. J., Klaiberg, A., & Paschke, R. (2004). Patient education and counseling, 53(3), 315-318. | Germany | 120 | 1. Computer-based hypoglycaemia education - adaptive version that can be personalised to patient needs 2. Computer-based hypoglycaemia education | NO | N=0 |
|  | Negarandeh, R., Mahmoodi, H., Noktehdan, H., Heshmat, R., & Shakibazadeh, E. (2013). Primary care diabetes, 7(2), 111-118. | Kurdistan | 135 | 1. Education using pictorial methods 2. Education using Teach back method (verbal, interactive) 3. Usual care | YES |  |
|  | Negri, C., Bacchi, E., Morgante, S., Soave, D., Marques, A., Menghini, E., ... & Moghetti, P. (2010). Diabetes Care, 33(11), 2333-2335. | Italy | 41 | 1. Supervised walking sessions plus counselling 2. Instructions aimed at encouraging physical activity | YES |  |
|  | Nelson, K., Taylor, L., Silverman, J., Kiefer, M., Hebert, P., Lessler, D., & Krieger, J. (2017). Preventing Chronic Disease, 14, E15. doi: 10.5888/pcd14.160344 | USA | 287 | 1. Community health worker diabetes self-management support  2. Usual care | NO | NK |
|  | Nesari, M., Zakerimoghadam, M., Rajab, A., Bassampour, S., & Faghihzadeh, S. (2010). Japan Journal of Nursing Science, 7(2), 121-128. | Iran | 61 | 1. 2x a week telephone counselling for 3 months after intensive education training 2. Control - education only. | YES |  |
|  | Ng, C. L., Goh, S. Y., Malhotra, R., Østbye, T., & Tai, E. S. (2010). Journal of physiotherapy, 56(3), 163-170. | Singapore | 60 | 1. Aerobic group 2. Resistance training group | NR |  |
|  | Nielsen, A. B., de Fine Olivarius, N., Gannik, D., Hindsberger, C., & Hollnagel, H. (2006). Diabetes Care, 29(5), 963-969. | Denmark | 1470 | 1. Personalised service and goal setting by GP 2. Usual care | YES |  |
|  | Nishita C, Cardazone G, Uehara DL, Tom T. Health Education & Behavior. 2012 40(5) 581–  591 | USA | 190 | 1. Life coaching and pharmacist services 2. Usual care | NR |  |
|  | Niswender, K., Piletic, M., Andersen, H., Conradsen Hiort, L., & Hollander, P. (2014). Diabetes, Obesity and Metabolism, 16(2), 186-192. | Argentina, Germany, Poland, Serbia, Slovakia, Slovenia, Spain, Turkey, USA | 611 | 1. Insulin detemir, dietary and exercise advice PLUS three face to face meetings and 3 phone calls with a dietician 2. Basic advice | NR |  |
|  | Noh, J. H., Cho, Y. J., Nam, H. W., Kim, J. H., Kim, D. J., Yoo, H. S., ... & Yoo, J. H. (2010). Diabetes Technology & Therapeutics, 12(5), 333-337. | South Korea | 40 | 1. Website and mobile phone support 2. Usual care | NR |  |
|  | O'Connor, P., Schmittdiel, J., Pathak, R., Harris, R., Newton, K. et al (2014). Diabetes care, 37-12, 3317-3324 | USA | 2378 | 1. Educational telephone calls (self-management intervention)  2. Usual care | NO | NK |
|  | Odnoletkova, I., Goderis, G., Nobels, F., Fieuws, S., Aertgeerts, B., Annemans, L., & Ramaekers, D. (2016). Diabetic Medicine, 33(6), 777-785. doi: 10.1111/dme.13092 | Belgium | 574 | 1. Target-driven telecoaching  2. Usual care | YES |  |
|  | Olry de Labry Lima, A., Bermúdez Tamayo, C., Pastor Moreno, G., Bolívar Muñoz, J., Ruiz Pérez, I., Johri, M., ... & Moratalla López, E. (2017). Gaceta Sanitaria, 31, 40-47. doi: 10.1016/j.gaceta.2016.05.017 | Spain | 184 | 1. Diabetes self-management record sheet (DSMRS) as part of the consultation  2. Standard care | YES |  |
|  | Olson, E. A., & McAuley, E. (2015). Journal of Behavioral Medicine, 38(6), 886-898. doi: 10.1007/s10865-015-9660-3 | USA | 125 | 1. Intervention group- exercise  2. Control group- education | NR |  |
|  | Orsama, A. L., Lähteenmäki, J., Harno, K., Kulju, M., Wintergerst, E., Schachner, H., ... & Fisher, W. A. (2013). Diabetes technology & therapeutics, 15(8), 662-669. | Finland | 48 | 1. Technology to monitor and report from home, receive feedback. Access their health care plan etc. 2. Usual care | YES |  |
|  | Pacaud, D., Kelley, H., Downey, A. M., & Chiasson, M. (2012). Journal of Diabetes, 36(5), 257-262. | Canada | 79 | 1. Web static education 2. Web interactive education 3. Control – face to face | NR |  |
|  | Partapsingh, V. A., Maharaj, R. G., & Rawlins, J. M. (2011). J Negat Results Biomed, 10, 13. | Trinidad | 122 | 1. Consultations based on stages of change theory 2. Usual care | NO | N=0 |
|  | Paschali, A. A., Goodrick, G. K., Kalantzi-Azizi, A., Papadatou, D., & Balasubramanyam, A. (2005). Perceptual and motor skills, 100(1), 61-68. | USA | 26 | 1. Exercise and counseling intervention with feedback information from accelerometer. 2. Exercise and counseling intervention without feedback information from accelerometer | NO | N=0 |
|  | Paz-Pacheco, E., Sandoval, M. A., Ardena, G. J., Paterno, E., Juban, N., Lantion-Ang, F. L., ... & Bongon, J. (2017). Primary health care research & development, 18(1), 35-49. doi:10.1017/S1463423616000335 | Philippines | 155 | 1. Intervention group- diabetes self-management education (DSME)  2. Control group- standard care | NO | N=0 |
|  | Peimani, M., Monjazebi, F., Ghodssi-Ghassemabadi, R., & Nasli- Esfahani, E. (2018). Patient education and Counseling, 101(3), 460-466. doi: 10.1016/j.pec.2017.10.007 | Iran | 200 | 1. Intervention group- peer support  2. Control group- usual care | YES |  |
|  | Peimani, M., Rambod, C., Omidvar, M., Larijani, B., Ghodssi- Ghassemabadi, R., Tootee, A., & Esfahani, E. N. (2016). Primary care diabetes, 10(4), 251-258. doi: 10.1016/j.pcd.2015.11.001 | Iran | 150 | 1. Individually tailored SMS  2. Non-tailored SMS  3. Control group | YES |  |
|  | Penalba, M., Moreno, L., Cobo, A., Reviriego, J., Rodríguez, A., Cleall, S., & Reaney, M. (2014). Endocrinología y Nutrición (English Edition), 61(10), 505-515. | Spain | 310 | 1. Intervention group- conversation maps  2. Control group- regular care | NR |  |
|  | Pereira, D. A., Costa, N. M. D. S. C., Sousa, A. L. L., Jardim, P. C. B. V., & Zanini, C. R. D. O. (2012). Revista latino-americana de enfermagem, 20(3), 478-485. | Brazil | 62 | 1. Educational intervention 2. Usual care | NR |  |
|  | Pérez-Escamilla, R., Damio, G., Chhabra, J., Fernandez, M. L., Segura-Pérez, S., Vega-López, S., ... & D’Agostino, D. (2015). Diabetes Care, 38, 197–205. doi: 10.2337/dc14-0327 | USA | 211 | 1. Intervention group- standard of care plus a 12-month-long, community health workers-led, culturally tailored diabetes education and counseling treatment  2. Control group- standard of care | YES |  |
|  | Petersen, K. S., Torpy, D. J., Chapman, I. M., Guha, S., Clifton, P. M., Turner, K., & Keogh, J. B. (2013). Appetite, 68, 147-151. | Australia | 78 | 1. Food education focusing on sodium levels on labels 2. Usual care | NR |  |
|  | Philis-Tsimikas, A., Fortmann, A., Lleva-Ocana, L., Walker, C., & Gallo, L. C. (2011). Diabetes care, 34(9), 1926-1931. | USA | 207 | 1. Self-management classes and subsequent monthly support groups, led by a trained peer educator. 2. Usual care | YES |  |
|  | Piatt, G. A., Songer, T. J., Brooks, M. M., Anderson, R. M., Simmons, D., Orchard, T. J., ... & Zgibor, J. C. (2011). Patient education and counseling, 82(2), 266-270. | USA | 119 | 1. Chronic Care Model (CCM) intervention 2. Provider education intervention 3. Usual care. | NR |  |
|  | Piette, J. D., Richardson, C., Himle, J., Duffy, S., Torres, T., Vogel, M., ... & Valenstein, M. (2011). Medical care, 49(7), 641. | USA | 339 | 1. Telephone-delivered cognitive behavioral therapy (CBT) and walking 2. Usual care | YES |  |
|  | Pimazoni-Netto, A., Rodbard, D., & Zanella on behalf of the Diabetes Education and Control Group, M. T. (2011). Diabetes technology & therapeutics, 13(10), 997-1004. | Brazil | 64 | 1. 7 clinic visits with 10hrs education 2. 3 clinic visits with 2hrs education | NO | N=4 |
|  | Plotnikoff, R. C., Karunamuni, N., Courneya, K. S., Sigal, R. J., Johnson, J. A., & Johnson, S. T. (2013). Annals of Behavioral Medicine, 45(1), 45-56. | Canada | 287 | 1. Stage readiness and seasonal written materials 2. As above and telephone counselling to promote physical activity 3. Usual care | NO | NK |
|  | Plotnikoff, R. C., Wilczynska, M., Cohen, K. E., Smith, J. J., & Lubans, D. R. (2017). Preventive medicine, 105, 404-411. 10.1016/j.ypmed.2017.08.027 | Australia | 84 | 1. eCoFit program- smartphone technology  2. Waitlist control group | NO | NK |
|  | Prezio, E. A., Cheng, D., Balasubramanian, B. A., Shuval, K., Kendzor, D. E., & Culica, D. (2013). Diabetes research and clinical practice, 100(1), 19-28. | USA | 180 | 1. Culturally tailored diabetes education program led by a community health worker 2. Usual care | NR |  |
|  | Quinn, C. C., Clough, S. S., Minor, J. M., Lender, D., Okafor, M. C., & Gruber-Baldini, A. (2008). Diabetes technology & therapeutics, 10(3), 160-168. | USA | 30 | 1. Well doc server, blue tooth glucose monitor, feedback and training 2. Usual care | NR |  |
|  | Quinn, C. C., Shardell, M. D., Terrin, M. L., Barr, E. A., Ballew, S. H., & Gruber-Baldini, A. L. (2011). Diabetes care, DC_110366. | USA | 213 | 1. Mobile phone coaching 2. Mobile phone coaching and website 3. Mobile phone coaching, website and decision making tool 4. Usual care | YES |  |
|  | Rachmani, R., Slavachevski, I., Berla, M., Frommer‐Shapira, R., & Ravid, M. (2005). Diabetic medicine, 22(4), 410-414. | Israel | 141 | 1. Patient participation and teaching programme 2. Usual care | NR |  |
|  | Ramanath, KV., Santhosh, Y.L.(2011). Asian Journal of Pharmaceutical and Clinical Research, 4(4), 15-20. | India | 100 | 1. Education face and face and written by pharmacist 2. Usual care | YES |  |
|  | Ramli, A. S., Selvarajah, S., Daud, M. H., Haniff, J., Abdul-Razak, S., Tg-Abu-Bakar, T. M. I., ... & Shafie, A. A. (2016). BMC Family Practice, 17(1), 157. doi: 10.1186/s12875-016-0557-1 | Malaysia | 888 | 1. EMPOWER Participatory Action Research  2. Usual care | NR |  |
|  | Reaney, M., Zorzo, E. G., Golay, A., Hermanns, N., Cleall, S., Petzinger, U., & Koivisto, V. (2013). Diabetes Spectrum, 26(4), 236-245. | Spain and Germany | 681 | 1. Conversation Map education tool group education sessions 2. Usual care | NR |  |
|  | Richardson, C. R., Mehari, K. S., McIntyre, L. G., Janney, A. W., Fortlage, L. A., Sen, A., ... & Piette, J. D. (2007). International Journal of Behavioral Nutrition and Physical Activity, 4(1), 59. | USA | 32 | 1. Automated Internet-based intervention using uploading-enhanced pedometers 2. Usual care | NO | NK |
|  | Rock, C. L., Flatt, S. W., Pakiz, B., Taylor, K. S., Leone, A. F., Brelje, K., ... & Sherwood, N. E. (2014). Diabetes care, 37(6), 1573-1580. | USA | 227 | 1. Low fat diet education 2. Low carb diet 3. Usual care | YES |  |
|  | Rodríguez-Idígoras, M. I., Sepúlveda-Muñoz, J., Sánchez-Garrido-Escudero, R., Martínez-González, J. L., Escolar-Castelló, J. L., Paniagua-Gómez, I. M., ... & Garófano-Serrano, D. (2009). Diabetes technology & therapeutics, 11(7), 431-437. | Spain | 347 | 1. Mobile phone, real time feedback consultation re glucose levels 2. Usual care | NR |  |
|  | Rosal, M. C., Ockene, I. S., Restrepo, A., White, M. J., Borg, A., Olendzki, B., ... & Reed, G. (2011). Diabetes care, 34(4), 838-844. | USA | 252 | 1. Latinos en control: intensive education 2. Usual care | NR |  |
|  | Rosal, M. C., Olendzki, B., Reed, G. W., Ockene, I., Gumieniak, O., & Scavron, J. (2005). Annals of Behavioral Medicine, 29(3), 225-235. | USA | 25 | 1. Individual counselling and groups that included multi-media educational presentations 2. Usual care | NR |  |
|  | Rosenbek Minet, L. K., Wagner, L., Lønvig, E. M., Hjelmborg, J., & Henriksen, J. E. (2011). Diabetologia, 54(7), 1620-1629. | Denmark | 349 | 1. Motivational interviewing counselling in year following education intervention 2. Usual care | YES |  |
|  | Rothschild, S.K., Martin, M.A., Swider, S.M., Lynas, C.T., Avery E.F., Janssen I., Powell, L.H. (2012) Contemporary Clinical Trials; 33: 369–377 | USA | 144 | 1. Home visits by community health worker (promotora) 2. Usual care | YES |  |
|  | Rubak S, Sandbæk A, Lauritzen T, Borch-Johnsen K, Christensen B. Scandinavian journal of primary health care. 2009 Jan 1;27(3):172-9 | Denmark | 234 | 1. Intensive diabetes treatment delivered by GPs trained in Motivational Interviewing 2. Intensive diabetes treatment | YES |  |
|  | Ruggiero, L., Moadsiri, A., Butler, P., Oros, S. M., Berbaum, M. L., Whitman, S., & Cintron, D. (2010). The Diabetes Educator, 36(1), 127-131. | USA | 100 | 1. Medical assistant coaching (MAC) group 2. Usual care | NR |  |
|  | Ruggiero, L., Riley, B. B., Hernandez, R., Quinn, L. T., Gerber, B. S., Castillo, A., ... & Butler, P. (2014). Western Journal of Nursing research, 36(9), 1052-1073. | USA | 270 | 1. Intervention group- culturally tailored medical assistant self-care coaching  2. Treatment as usual | YES |  |
|  | Ruiz JG, Andrade AD, Anam R, Lisigurski M, Karanam C, Sharit J. The Diabetes Educator. 2013 Oct 29:0145721713508825. | USA | 166 | 1. Computer based programmed instruction with dynamic avatar 2. Computer based programmed instruction with voice 3. Computer based programmed instruction with static avatar 4. Computer based programmed instruction with text and dynamic avatar 5. Computer based programmed instruction with text (control) | YES |  |
|  | Rygg, L. Ø., Rise, M. B., Grønning, K., & Steinsbekk, A. (2012). Patient education and counseling, 86(1), 98-105. | Norway | 146 | 1. Interactive DSME group 2. Waiting list control | NR |  |
|  | Sacco, W. P., Bykowski, C. A., Mayhew, L. L., & White, K. E. (2012). Diabetes research and clinical practice, 95(1), 62-67. | USA | 62 | 1. Brief telephone lifestyle modiﬁcation program 2. Usual care | YES |  |
|  | Safford, M. M., Andreae, S., Cherrington, A. L., Martin, M. Y., Halanych, J., Lewis, M., ... & Richman, J. S. (2015). The Annals of Family Medicine, 13(Suppl 1), S18-S26. | USA | 424 | 1. Diabetes education class, personalized diabetes report card, paired with peer coaches and telephone interactions  2. Diabetes education class and a personalized diabetes report card | NR |  |
|  | Safren, SA, Gonzalez, JS, Wexler, DJ, Psaros, C, Delahanty, LM, Blashill, AJ, Margolina, AI, and Cagliero, C. (2014). Diabetes care, 37:625-633 | USA | 87 | 1. Enhanced treatment as usual and CBT 2. Enhanced treatment as usual only | YES |  |
|  | Salinero-Fort, M. A., Pau, E. C., Arrieta-Blanco, F. J., Abanades-Herranz, J. C., Martín-Madrazo, C., Rodés-Soldevila, B., & de Burgos-Lunar, C. (2011). BMC public health, 11(1), 267. | Spain | 608 | 1. PRECEDE educational intervention 2. Conventional education | NR |  |
|  | Samtia, A. M., Rasool, M. F., Ranjha, N. M., Usman, F., & Javed, I. (2013). Tropical Journal of Pharmaceutical Research, 12(5), 851-856. | Pakistan | 348 | 1. Pharmacist-led multifactorial intervention 2. Usual care | NR |  |
|  | Samuel-Hodge, C. D., Keyserling, T. C., France, R., Ingram, A. F., Johnston, L. F., Davis, L. P., ... & Cole, A. S. (2006). Preventing Chronic Disease, 3(3). | USA | 204 | 1. Culturally sensitive self-management education, individual, groups, postcards and telephone 2. Minimal intervention | NR |  |
|  | Sarayani, A., Mashayekhi, M., Nosrati, M., Jahangard-Rafsanjani, Z., Javadi, M., Saadat, N., ... & Gholami, K. (2018). International journal of clinical pharmacy, 40(2), 345-353. doi: 10.1007/s11096-018-0593-0 | Iran | 100 | 1. Telephone-based intervention  2. Usual care | NO | NK |
|  | Sardar MA, Boghrabadi V, Sohrabi M, Aminzadeh R, Jalalian M. Global journal of health science. 2014 Mar;6(2):196. | Iran | 53 | 1. Group exercise training 2. Usual care | YES |  |
|  | Sarkadi, A., & Rosenqvist, U. (2004). Patient education and counseling, 53(3), 291-298. | Sweden | 77 | 1. Group educational program led by specially trained pharmacists, assisted by a diabetes nurse specialist 2. Waiting list control | NR |  |
|  | Saslow LR, Kim S, Daubenmier JJ, Moskowitz JT, Phinney SD, Goldman V, Murphy EJ, Cox RM, Moran P, Hecht FM. PloS one. 2014 Apr 9;9(4):e91027. | USA | 35 | 1. Medium carb diet educational groups 2. Ketogenic diet educational groups | YES |  |
|  | Sazlina, S. G., Browning, C. J., & Yasin, S. (2015). Frontiers in Public Health, 3, 178. | Malaysia | 69 | 1. Intervention group- personalised feedback  2. Intervention group- peer support  3. Usual care | YES |  |
|  | Scain, S. F., Friedman, R., & Gross, J. L. (2009). The Diabetes Educator, 35(4), 603-611. | Brazil | 104 | 1. Structured group education program delivered by a trained nurse educator 2. Usual care | YES |  |
|  | Sevick, M. A., Korytkowski, M., Stone, R. A., Piraino, B., Ren, D., Sereika, S., ... & Burke, L. E. (2012). Journal of the Academy of Nutrition and Dietetics, 112(8), 1147-1157. | USA | 296 | 1. Group counseling sessions guided by Social Cognitive Theory 2. Usual care | NR |  |
|  | Sevick, M. A., Zickmund, S., Korytkowski, M., Piraino, B., Sereika, S., Mihalko, S., ... & Marsh, R. (2008). Contemporary clinical trials, 29(3), 396-409. | USA | 151 | 1. SCT-based behavioral intervention was paired with Personal digital assistant based self-monitoring 2. Attention control group | NR |  |
|  | Shahid, M., Mahar, S. A., Shaikh, S., & Shaikh, Z. U. (2015). J Coll Physicians Surg Pak, 25(3), 166-71. | Pakistan | 440 | 1. Telephone monitoring and 4 month physical examination  2. Examined initially and after 4 months physically in the clinic | YES |  |
|  | Shakibazadeh, E., Bartholomew, L. K., Rashidian, A., & Larijani, B. (2015). Health promotion international, 31(3), 623-634. doi: 10.1093/heapro/dav006 | Iran | 280 | 1. Persian Diabetes Self-Management Education  2. Usual care | NR |  |
|  | Sharifirad, G., Najimi, A., Hassanzadeh, A., & Azadbakht, L. (2013). Journal of diabetes, 5(2), 157-162. | Iran | 100 | 1. Tailored face to face nutritional education 2. Usual care | NR |  |
|  | Sharoni, S. K. A., Rahman, H. A., Minhat, H. S., Shariff-Ghazali, S., & Ong, M. H. A. (2018). PloS one, 13(3), e0192417. doi: 10.1371/journal.pone.0192417 | Malaysia | 76 | 1. Health education program on foot self-care behaviour  2. Standard care | YES |  |
|  | Sherifali, D., Greb, J. L., Amirthavasar, G., Hunt, D., Haynes, R. B., Harper, W., ... & Pullenayegum, E. (2011). Diabetes care, 34(8), 1794-1798. | Canada | 465 | 1. Computer based tailored feedback to promote self-management 2. Usual care | NR |  |
|  | Shetty, A. S., Chamukuttan, S., Nanditha, A., Raj, R. K., & Ramachandran, A. (2011). J Assoc Physicians India, 59, 711-714. | India | 215 | 1. SMS messages. 2. Usual care | NR |  |
|  | Shi, M., Liu, Z. L., Zhu, Y. B., Xu, M. Y., Duan, X. Y., Shi, H. M., ... & Yu, X. H. (2018). Chinese Journal of Integrative Medicine, 24(2), 94-102. doi: 10.1007/s11655-015-2113-6 | China and Japan | 256 | 1. Integrative education  2. Usual education | NR |  |
|  | Shi, Q., Ostwald, S. K., & Wang, S. (2010). Journal of clinical nursing, 19(3‐4), 398-404. | China | 157 | 1. One-month hospital-based clinic intervention 2. Usual care | NR |  |
|  | Shibayama, T., Kobayashi, K., Takano, A., Kadowaki, T., & Kazuma, K. (2007). Diabetes research and clinical practice, 76(2), 265-268. | Japan | 134 | 1. One-to-one counselling with a Certiﬁed Expert Nurse (CEN) in diabetes nursing 2. Usual care | NO | NK |
|  | Siebolds, M., Gaedeke, O., Schwedes, U., & SMBG Study Group. (2006). Patient education and counseling, 62(1), 104-110. | Germany and Austria | 223 | 1. Diet plus oral antidiabetic medication using an SMBG device 2. Diet alone | NR |  |
|  | Silfee, V., Petosa, R., Laurent, D., Schaub, T., & Focht, B. (2016). Psychology, Health & Medicine, 21(6), 715-723. doi: 10.1080/13548506.2016.1139144 | USA | 24 | 1. Behavioural intervention on dimensions of self-regulation and physical activity  2. Information regarding physical activity habits | NR |  |
|  | Silva, D. D. R., & Bosco, A. A. (2015). Diabetology & metabolic syndrome, 7(1), 2. | Brazil | 23 | 1. 6-week Educational program  2. 2-week Educational program | YES |  |
|  | Siminerio, L., Ruppert, K. M., & Gabbay, R. A. (2013). The Diabetes Educator, 39(5), 705-713. | USA | 141 | Post education diabetes self-management support delivered by   1. Certified diabetes educators 2. Peer support 3. Office staff 4. Usual care | NR |  |
|  | Simmons D, Gamble GD, Foote S, Cole DR, Coster G. Diabetic medicine. 2004 Mar 1;21(3):214-7. | New Zealand | 398 | 1. Passport containing information on diabetes and interaction with health professionals 2. Control booklet | YES |  |
|  | Simmons, D., Cohn, S., Bunn, C., Birch, K., Donald, S., Paddison, C., ... & Graffy, J. (2013). BMC family practice, 14(1), 5. | UK | 61 | 1. Group-based peer support program 2. One-to-one support 3. a combination of both group and one-to-one support 4. Control group – usual care | YES |  |
|  | Sinclair, K. A., Makahi, E. K., Shea-Solatorio, C., Yoshimura, S. R., Townsend, C. K., & Kaholokula, J. K. (2013). Annals of Behavioral Medicine, 45(1), 24-32. | USA | 82 | 1. Diabetes self-management educational intervention -Partners in Care 2. Waiting list control | YES |  |
|  | Skelly, A. H., Carlson, J., Leeman, J., Soward, A., & Burns, D. (2009). Nursing research, 58(6), 410. | USA | 180 | 1. Symptom-focused intervention involved teaching and counseling modules delivered by a nurse in the participant’s home 2. As above with telephone support 3. Usual care | NR |  |
|  | Smith, S., Bury, G., O'leary, M., Shannon, W., Tynan, A., Staines, A., & Thompson, C. (2004). Family Practice, 21(1), 39-45. | Ireland | 183 | 1. Training for diabetes nurses to reinforce nutritional info 2. Usual care | NO | NK |
|  | Sone, H., Tanaka, S., Iimuro, S., Oida, K., Yamasaki, Y., Oikawa, S., ... & Yoshimura, Y. (2010). Diabetologia, 53(3), 419-428. | Japan | 2033 | 1. Education on lifestyle modification regarding dietary habits, physical activities and adherence to treatment by telephone counselling and at each outpatient clinic visit 2. Usual care | NO | N=0 |
|  | Song MS, Kim HS. Applied Nursing Research. 2009 Feb 28;22(1):42-7. | South Korea | 49 | 1. Multidisciplinary education, complication monitoring and telephone counselling. 2. Usual care | YES |  |
|  | Sönnichsen AC, Winkler H, Flamm M, Panisch S, Kowatsch P, Klima G, Fürthauer B, Weitgasser R. BMC family practice. 2010 Nov 5;11(1):1. | Austria | 1489 | 1. Patient group education 2. Usual care | YES |  |
|  | Sorkin, D. H., Mavandadi, S., Rook, K. S., Biegler, K. A., Kilgore, D., Dow, E., & Ngo-Metzger, Q. (2014). Health Psychol, 33(6), 566-575. | USA | 89  dyads | 1. Mother and daughters receiving diet and self-management information – groups, home visits, telephone support 2. Control: educational materials | NO | NK |
|  | Spencer, M. S., Rosland, A. M., Kieffer, E. C., Sinco, B. R., Valerio, M., Palmisano, G., ... & Heisler, M. (2011). American Journal of Public Health, 101(12), 2253-2260. | USA | 183 | 1. Culturally tailored, behavioral theory–based community health worker intervention 2. Waiting list control | NO | NR |
|  | Steed, L., Lankester, J., Barnard, M., Earle, K., Hurel, S., & Newman, S. (2005). Journal of health psychology, 10(2), 261-276. | UK | 124 | 1. UCL-DSMP - group-based self-management programme. 2. Usual care | YES |  |
|  | Stone, R. A., Rao, R. H., Sevick, M. A., Cheng, C., Hough, L. J., Macpherson, D. S., ... & DeRubertis, F. R. (2010). Diabetes care, 33(3), 478-484. | USA | 150 | 1. Diabetes management support intervention using the Viterion 100 Monitor home telemonitoring device plus monthly calls for education and review. 2. Monthly calls | NR |  |
|  | Sturt, J. A., Whitlock, S., Fox, C., Hearnshaw, H., Farmer, A. J., Wakelin, M., ... & Dale, J. (2008). Diabetic Medicine, 25(6), 722-731. | UK | 244 | 1. Diabetes Manual trial 2. 6-month deferred intervention | NR |  |
|  | Sung, K., & Bae, S. (2012). Nursing & health sciences, 14(4), 438-445. | South Korea | 40 | 1. 24 week walking programme and diet education 2. Usual care | NR |  |
|  | Suppapitiporn, S., Chindavijak, B., & Onsanit, S. (2005). Journal-Medical Association of Thailand, 88, S134. | Thailand | 360 | 1. Counselling by pharmacist 2. 2. Plus special containers. 3. 3. Plus booklet. 4. 4. Plus everything | NR |  |
|  | Suriyawongpaisal, P., Tansirisithikul, R., Sakulpipat, T., Charoensuk, P., & Aekplakorn, W. (2016). Journal of the Medical Association of Thailand, 99(2), 125-132. | Thailand | 191 | 1. 7-point self-monitoring of blood glucose (SMBG)  2. 5-point SMBG  3. Usual care | NO | NK |
|  | Surucu, H., A., Kizilci, S., & Ergor, G. (2017). International Journal of Caring Sciences, 10(1), 479-489. | Turkey | 139 | 1. Intervention group  2. Control group | NR |  |
|  | Swoboda, C. M., Miller, C. K., & Wills, C. E. (2017). Patient Education and Counseling, 100(7), 1367-1373. doi: 10.1016/j.pec.2017.02.007 | USA | 54 | 1. 16-week decision support and goal-setting (Multiple goal (MG) intervention & single goal (SG) intervention)  2. Attention control | NO | N=0 |
|  | Taggart, L., Truesdale, M., Carey, M. E., Martin‐Stacey, L., Scott, J., Bunting, B., ... & Clarke, J. M. (2018). Diabetic Medicine, 35(1), 137-146. doi: 10.1111/dme.13539 | UK | 39 | 1. Diabetes education and self-management for ongoing and newly diagnosed (DESMOND) intervention group  2. Usual care control group | NO | NK |
|  | Tan, M. Y., Magarey, J. M., Chee, S. S., Lee, L. F., & Tan, M. H. (2011). Health education research, 26(5), 896-907. | Malaysia | 164 | 1. Face to face and telephone call education 2. Usual care | YES |  |
|  | Tang, T. S., Funnell, M. M., Sinco, B., Spencer, M. S., & Heisler, M. (2015). The Annals of Family Medicine, 13(Suppl 1), S27-S35. | USA | 106 | 1. Diabetes self-management education (DSME) program plus peer support intervention  2. DSME | YES |  |
|  | Tang, T. S., Funnell, M., Sinco, B., Piatt, G., Palmisano, G., Spencer, M. S., ... & Heisler, M. (2014). Diabetes care, 37(6), 1525- 1534. doi:10.2337/dc13-2161 | USA | 116 | 1. Peer leader intervention  2. Community health worker intervention | NR |  |
|  | Tavakolizadeh J, Moghadas M, Ashraf H. Iranian red crescent medical journal. 2014 Apr;16(4). | Iran | 60 | 1. Self-regulation education sessions and book 2. Usual care | YES |  |
|  | Taveira, T. H., Dooley, A. G., Cohen, L. B., Khatana, S. A. M., & Wu, W. C. (2011). Annals of Pharmacotherapy, 45(11), 1346-1355. | USA | 88 | 1. Multidisciplinary education and pharmacist-led intensive behavioral and pharmacologic group 2. Usual care | YES |  |
|  | Taveira, T. H., Friedmann, P. D., Cohen, L. B., Dooley, A. G., Khatana, S. A. M., Pirraglia, P. A., & Wu, W. C. (2010). The Diabetes Educator, 36(1), 109-117. | USA | 118 | 1. Multidisciplinary education and pharmacist-led intensive behavioral and pharmacologic group 2. Usual care | YES |  |
|  | Taylor, J. D., Fletcher, J. P., & Tiarks, J. (2009). Physical therapy, 89(9), 884-892. | USA | 24 | 1. Physical therapist– directed exercise counseling plus ﬁtness center– based exercise training 2. Laboratory-based, supervised exercise | NR |  |
|  | Taylor, K. I., Oberle, K. M., Crutcher, R. A., & Norton, P. G. (2005). Biological Research for Nursing, 6(3), 207-215. | Canada | 39 | 1. 4 to 5 visits from the nurse and 1 visit from the dietitian. 2. Usual care | NR |  |
|  | Thom, D. H., Ghorob, A., Hessler, D., De Vore, D., Chen, E., & Bodenheimer, T. A. (2013). The Annals of Family Medicine, 11(2), 137-144. | USA | 299 | 1. Peer coaching face to face and telephone 2. Usual care | YES |  |
|  | Thom, D. H., Hessler, D., Willard-Grace, R., Bodenheimer, T., Najmabadi, A., Araujo, C., & Chen, E. H. (2014). Patient education and Counseling, 96(1), 135-138. | USA | 441 | 1. Health coaching face to face and telephone 2. Usual care | NR |  |
|  | Tildesley, H. D., Mazanderani, A. B., & Ross, S. A. (2010). Diabetes Care, 33(8), 1738-1740. | Canada | 47 | 1. Web site for review and receipt of feedback from endocrinologist 2. Usual care | NR |  |
|  | Toobert, D. J., Strycker, L. A., Barrera Jr, M., & Glasgow, R. E. (2010). American journal of health behavior, 34(6), 680. | USA | 279 | 1. Weekly meetings, mediterranean diet, physical activity 2. Usual care | YES |  |
|  | Toobert, D. J., Strycker, L. A., King, D. K., Barrera Jr, M., Osuna, D., & Glasgow, R. E. (2011). Translational behavioral medicine, 1(3), 416-426. | USA | 280 | 1. Mediterranean diet, lifestyle education retreat and group meetings 2. Enhanced usual care | YES |  |
|  | Torbjørnsen, A., Jenum, A. K., Småstuen, M. C., Årsand, E., Holmen, H., Wahl, A. K., & Ribu, L. (2014). JMIR mHealth and uHealth, 2(4). doi: 10.2196/mhealth.3535  Holmen, H., Torbjørnsen, A., Wahl, A. K., Jenum, A. K., Småstuen, M. C., Årsand, E., & Ribu, L. (2014). JMIR mHealth and uHealth, 2(4), e57. doi: 10.2196/mhealth.3882 | Norway | 164 | 1. Mobile phone–based self-management system Few Touch Application (FTA)  2. FTA plus health counselling  3. Usual care | YES |  |
|  | Trento, M., Basile, M., Borgo, E., Grassi, G., Scuntero, P., Trinetta, A., ... & Porta, M. (2008). Journal of endocrinological investigation, 31(11), 1038-1042. | Italy | 49 | 1. Group care administered by nurses 2. Usual care | NR |  |
|  | Trento, M., Gamba, S., Gentile, L., Grassi, G., Miselli, V., Morone, G., ... & Cavallo, F. (2010). Diabetes care, 33(4), 745-747. | Italy | 795 | 1. Group education 2. Individual consultation | NR |  |
|  | Trento, M., Passera, P., Borgo, E., Tomalino, M., Bajardi, M., Cavallo, F., & Porta, M. (2004). Diabetes care, 27(3), 670-675. | Italy | 120 | 1. Group education 2. Individual consultation | NR |  |
|  | Trief, P. M., Izquierdo, R., Eimicke, J. P., Teresi, J. A., Goland, R., Palmas, W., ... & Weinstock, R. S. (2013). Ethnicity & health, 18(1), 83-96. | USA | 1665 | 1. Telemedicine case management (televideo educator visits, individualized goal-setting/problem solving) 2. Usual care. | NO | NR |
|  | Trief, P., Sandberg, J. G., Ploutz-Snyder, R., Brittain, R., Cibula, D., Scales, K., & Weinstock, R. S. (2011). Families, Systems, & Health, 29(3), 253. | USA | 44 | 1. Work book and telephone interventions/support - couples 2. Individuals 3. Enhanced usual care | NR |  |
|  | Tsang, T., Orr, R., Lam, P., Comino, E. J., & Singh, M. F. (2007). interventions in aging, 2(3), 429. | Australia | 38 | 1. Tai chi 2. Sham exercise | YES |  |
|  | Tucker, C. M., Lopez, M. T., Campbell, K., Marsiske, M., Daly, K., Nghiem, K., ... & Patel, A. (2014). Journal of health care for the poor and underserved, 25(1), 292. | USA | 130 | 1. Workshops that were culturally sensitive 2. Waiting list control | NR |  |
|  | Tuthill, A., Quinn, A., McColgan, D., McKenna, M., O’Shea, D., & McKenna, T. J. (2007). Diabetes, Obesity and Metabolism, 9(6), 917-919. | Ireland | 68 | 1. Group sessions dietary advice from a dietician and exercise advice from physiotherapist, 2. Usual care | NR |  |
|  | Tutino, G. E., Yang, W. Y., Li, X., Li, W. H., Zhang, Y. Y., Guo, X. H., ... & So, W. Y. (2017). Diabetic Medicine, 34(3), 440-450. doi: 10.1111/dme.13164 | China | 3586 | 1. Diamond group- Diabetes Monitoring Database  2. Jade group- Joint Asia Diabetes Evaluation | NR |  |
|  | Utz SW, Williams IC, Jones R, Hinton I, Alexander G, Yan G, Moore C, Blankenship J, Steeves R, Oliver MN. The Diabetes Educator. 2008 Sep 1;34(5):854-65. | USA | 21 | 1. Individual education culturally tailored for rural African Americans 2. Group education culturally tailored for rural African Americans. | YES |  |
|  | Vadstrup, E. S., Frølich, A., Perrild, H., Borg, E., & Røder, M. (2011). Patient education and counseling, 84(2), 185-190. | Denmark | 143 | 1. Multidisciplinary group based rehabilitation programme 2. Individual counselling programme | YES |  |
|  | Vaishali, K., Kumar, K. V., Adhikari, P., & UnniKrishnan, B. (2012). Physical & Occupational Therapy in Geriatrics, 30(1), 22-30. | India | 57 | 1. Education plus yoga 2. Education | NR |  |
|  | Van der Wulp, I., de Leeuw, J. R. J., Gorter, K. J., & Rutten, G. E. H. M. (2012). Diabetic medicine, 29(10), e390-e397. | Netherlands | 133 | 1. Peer led self-management coaching programme 2. Usual care | NR |  |
|  | van Dijk-de Vries, A., van Bokhoven, M. A., Winkens, B., Terluin, B., Knottnerus, J. A., van der Weijden, T., & van Eijk, J. T. M. (2015). BMJ open, 5(6), e007014. | Netherlands | 264 | 1. Self-Management Support (SMS) delivered by practice nurses  2. Usual care | NO | NK |
|  | Van Dyck, D., De Greef, K., Deforche, B., Ruige, J., Bouckaert, J., Tudor-Locke, C. E., ... & De Bourdeaudhuij, I. (2013). Health education research, 28(3), 539-545. | Belgium | 92 | 1. Face to face and telephone support 2. Usual care | NR |  |
|  | Van Rooijen AJ, Rheeder P, Eales CJ, Becker PJ. Quarterly Journal of Medicine. 2004 Jun 1;97(6):343-51. | South Africa | 157 | 1. Supervised exercise with homework 2. Relaxation classes | YES |  |
|  | Van Rooijen, A. J., Viviers, C. M., & Becker, P. J. (2010). South African Journal of Physiotherapy, 66(2), 9-16. | South Africa | 51 | 1. Group classes – education, nutrition self-management and physical activity 2. Usual care | YES |  |
|  | Van Sluijs, E. M. F., Van Poppel, M. N. M., Twisk, J. W. R., Brug, J., & Van Mechelen, W. (2005). Health education research, 20(3), 345-356. | Netherlands | 771 | 1. PACE (Physician-based Assessment and Counseling for Exercise) 2. Usual care and standard information | NR |  |
|  | van Sluijs, E. M., van Poppel, M. N., Twisk, J. W., Paw, M. J. C. A., Calfas, K. J., & Van Mechelen, W. (2005). American Journal of Public Health, 95(10), 1825. | Netherlands | 400 | 1. Exercise intervention 2. Usual care | NO | NK |
|  | Varney, J. E., Weiland, T. J., Inder, W. J., & Jelinek, G. A. (2014). Internal medicine journal, 44(9), 890-897. doi:10.1111/imj.12515 | Australia | 94 | 1.Intervention group- usual diabetes care plus 6 months of telephone coaching  2. Usual care control group | NR |  |
|  | Venmans LM, Gorter KJ, Baard KP, Rutten GE, Hak E. 2007 Sep 30;1(3):135-42. | Netherlands | 200 | 1. Educational leaflet 2. Usual care | YES |  |
|  | Venmans, L. M., Gorter, K. J., Hak, E., & Rutten, G. E. (2008). Diabetes Care, 31(3), 402-407. | Netherlands | 1124 | 1. Group meeting, leaflet, web site, consultation 2. Usual care | YES |  |
|  | Vervloet, M., van Dijk, L., Santen-Reestman, J., Van Vlijmen, B., Van Wingerden, P., Bouvy, M. L., & de Bakker, D. H. (2012). International journal of medical informatics, 81(9), 594-604. | Netherlands | 415 | 1. Real time medication monitoring: smart pill boxes, sms and web access 2. Real time medication monitoring. 3. Usual care | NO | NK |
|  | Wakefield BJ, Holman JE, Ray A, Scherubel M, Adams MR, Hillis SL, Rosenthal GE. Telemedicine and e-Health. 2011 May 1;17(4):254-61. | USA | 302 | 1. Remote monitoring high intensity 2. remote monitoring low intensity 3. Usual care | YES |  |
|  | Waki, K., Fujita, H., Uchimura, Y., Omae, K., Aramaki, E., Kato, S., ... & Ohe, K. (2014). Journal of diabetes science and technology, 8(2), 209-215. | Japan | 54 | 1. DialBetics - Smartphone-based Self-management Support System 2. Non-DialBetics | NR |  |
|  | Walker, E. A., Shmukler, C., Ullman, R., Blanco, E., Scollan-Koliopoulus, M., & Cohen, H. W. (2011). Diabetes Care, 34(1), 2-7. | USA | 526 | 1. A tailored telephonic education intervention 2. A print education intervention | NO | NK |
|  | Wattana, C., Srisuphan, W., Pothiban, L., & Upchurch, S. L. (2007). Nursing & health sciences, 9(2), 135-141. | Thailand | 147 | 1. Self-management programme 2. Usual care | NR |  |
|  | Wattanakorn, K.; Deenan, A.; Puapan, S.; Scheider, J.K. (2013)Pacific Rim Inrernational Journal of Nursing Research. 17(4).356-369. | Thailand | 76 | 1. Individual sessions MI and Illness Perceptions 2. Health education | YES |  |
|  | Wayne, N., Perez, D. F., Kaplan, D. M., & Ritvo, P. (2015). Journal of Medical Internet Research, 17(10). | Canada | 97 | 1. Health coaching with phone monitoring  2. Health coaching | NO | NK |
|  | Welch G, Zagarins SE, Feinberg RG, Garb JL. Diabetes research and clinical practice. 2011 Jan 31;91(1):54-60. | USA | 234 | 1. MI and diabetes self-management education 2. Education alone 3. MI, education and computerized summary 4. Education and computerized summary | YES |  |
|  | West, D. S., DiLillo, V., Bursac, Z., Gore, S. A., & Greene, P. G. (2007). Diabetes care, 30(5), 1081-1087. | USA | 217 | 1. Weight control classes and MI 2. Weight control classes | NR |  |
|  | Weymiller, A. J., Montori, V. M., Jones, L. A., Gafni, A., Guyatt, G. H., Bryant, S. C., ... & Smith, S. A. (2007). Archives of internal medicine, 167(10), 1076-1082. | USA | 98 | 1. Statin Choice decision 2. Mayo Clinic standard edu­cational pamphlet about cholesterol management. | NO | NK |
|  | Whitehead, L. C., Crowe, M. T., Carter, J. D., Maskill, V. R., Carlyle, D., Bugge, C., & Frampton, C. M. (2017). Journal of evaluation in clinical practice, 23(4), 821-829. doi: 10.1111/jep.12725 | New Zealand | 157 | 1. Intervention group - education  2. Intervention group - education plus Acceptance and Commitment therapy  3. Control group - usual care | NO | N=1 |
|  | Whittemore, R., Melkus, G. D., Sullivan, A., & Grey, M. (2003). The Diabetes Educator, 30(5), 795-804. | USA | 53 | 1. Multifaceted nurse-coaching intervention, face to face and phone 2. Usual care | NR |  |
|  | Wichit, N., Mnatzaganian, G., Courtney, M., Schulz, P., & Johnson, M. (2017). Diabetes Research and Clinical Practice, 123, 37-48. doi: 10.1016/j.diabres.2016.11.013 | Thailand | 140 | 1. Intervention group- routine care + a family-oriented, carersupported diabetes self-management program  2. Control group- routine care | NO | NK |
|  | Williams A, Manias E, Walker R, Gorelik A.. Journal of advanced nursing. 2012 Nov 1;68(11):2515-25. | Australia | 120 | 1. Training and then telehealth report, interactive, DVD 2. Usual care | YES |  |
|  | Williams, G. C., Lynch, M., & Glasgow, R. E. (2007). Health Psychology, 26(6), 728. | USA | 886 | 1. Patient-centered, computer-assisted diabetes care intervention 2. Computer questionnaires | NR |  |
|  | Williams, G. C., McGregor, H., Zeldman, A., Freedman, Z. R., Deci, E. L., & Elder, D. (2005). Patient education and counseling, 56(1), 28-34. | USA | 232 | 1. Three activation sessions 2. Watched three educational videos prior to subsequent practitioner visits | NR |  |
|  | Wing, R. Look AHEAD Research Group. (2010). Archives of internal medicine, 170(17), 1566.  Knowler, W., Bahnson, J., Bantle, J., Bertoni, A. et al (2014). Lancet Diabetes Endocrinology, 2(10), 801-809.  Breyer, B., Phelan, S., Hogan, P., Rosen, R et al (2014). Journal of Urology, 192(1), 144-149.  Gallagher, D., Heshka, S., Kelley, D., Thornton, J., Boxt, L., Sunyer, F., Patricio, J., Mancino, J., Clark, J. (2014). Diabetes Care, 37(12), 3325-3332.  Alonso, A.,Bahnson, J et al. (2015). American Heart Journal, 170(4), 770-777.  The Look AHEAD Research Group. (2017). Diabetologia, 60(6), 980–988.doi: 10.1007/s00125-017-4253-z | USA | 5145 | 1. Intervention group- intensive lifestyle intervention  2. Control group- diabetes support and education | YES |  |
|  | Wisse, W., Rookhuizen, M. B., de Kruif, M. D., van Rossum, J., Jordans, I., ten Cate, H., ... & Meesters, E. W. (2010). Diabetes research and clinical practice, 88(2), e10-e13. | Netherlands | 74 | 1. Structured and personalized exercise prescription 2. Usual care | YES |  |
|  | Wolever RQ, Dreusicke M, Fikkan J, Hawkins TV, Yeung S, Wakefield J, Duda L, Flowers P, Cook C, Skinner E. The Diabetes Educator. 2010 Jul 1;36(4):629-39. | USA | 56 | 1. Integrative health coaching 2. Usual care | YES |  |
|  | Wolf, A. M., Conaway, M. R., Crowther, J. Q., Hazen, K. Y., Nadler, J. L., Oneida, B., & Bovbjerg, V. E. (2004). Diabetes care, 27(7), 1570-1576. | USA | 147 | 1. Lifestyle case management plus education 2. Usual care | NR |  |
|  | Wolf, M. S., Seligman, H., Davis, T. C., Fleming, D. A., Curtis, L. M., Pandit, A. U., ... & DeWalt, D. A. (2013). Journal of General Internal Medicine, 29(1), 59-67. | USA | 486 | 1. Diabetes Guide and brief counseling - In-house: face to face and phone 2. Diabetes Guide and brief counseling - Outsourced: phone support | NO | NR |
|  | Wu, C. J. J., Chang, A. M., Courtney, M., & Kostner, K. (2012). International nursing review, 59(3), 345-352. | Australia | 28 | 1. Peer support diabetes education 2. Usual education | NR |  |
|  | Wu, C. J., Sung, H. C., Chang, A. M., Atherton, J., Kostner, K., & McPhail, S. M. (2017). Nursing & health sciences, 19(3), 307-315. doi: doi: 10.1111/nhs.12346 | Australia and Taiwan | 181 | 1. Cardiac-diabetes self-management program 2. Educational information | NR |  |
|  | Wu, L., Forbes, A., & While, A. (2010). Journal of Telemedicine and Telecare, 16(4), 221-223. | UK | 46 | 1. A telephone booster intervention 2. Usual care | YES |  |
|  | Wu, S. F. V., Liang, S. Y., Wang, T. J., Chen, M. H., Jian, Y. M., & Cheng, K. C. (2011). Journal of clinical nursing, 20(17‐18), 2655-2665. | Taiwan | 145 | 1. Diabetes educational programme AND self-management, booklet, DVD, telephone support 2. Diabetes educational programme | NR |  |
|  | Yannakoulia, M., Poulia, K. A., Mylona, E., & Kontogianni, M. D. (2007). Rev Diabet Stud, 4(4), 226-230. | Greece | 30 | 1. Goal orientated face to face lifestyle intervention 2. Usual care | NO | N=0 |
|  | Yoo, H. J., Park, M. S., Kim, T. N., Yang, S. J., Cho, G. J., Hwang, T. G., ... & Choi, K. M. (2009). Diabetic Medicine, 26(6), 628-635. | South Korea | 123 | 1. Telehealth: mobile phone reporting of Ac and weight and immediate feedback 2. Usual care | NR |  |
|  | Yoon, K. H., & Kim, H. S. (2008). Diabetes research and clinical practice, 79(2), 256-261. | South Korea | 51 | 1. Telehealth: mobile phone reporting of Ac and weight and immediate feedback by SMS 2. Usual care | NR |  |
|  | Young, L. A., Buse, J. B., Weaver, M. A., Vu, M. B., Mitchell, C. M., Blakeney, T., ... & Donahue, K. E. (2017). JAMA internal medicine, 177(7), 920-929. doi:10.1001/jamainternmed.2017.1233 | USA | 450 | 1.No self monitoring of blood glucose (SMBG)  2. SMBG, no messaging  3.SMBG, with enhanced messaging | NO | NK |
|  | Young, R. J., Taylor, J., Friede, T., Hollis, S., Mason, J. M., Lee, P., ... & Gibson, J. M. (2005). Diabetes care, 28(2), 278-282. | UK | 591 | 1. Call center. Trained non-medical staff 2. Usual care | NR |  |
|  | Yuan, C., Lai, C. W., Chan, L. W., Chow, M., Law, H. K., & Ying, M. (2014). Journal of diabetes research, 2014. doi: 10.1155/2014/789761 | China | 88 | 1. Self-management education  2. Standard advice on medical nutrition  therapy | YES |  |
|  | Zhou, W., Chen, M., Yuan, J., & Sun, Y. (2016). Diabetes Research and Clinical Practice, 116, 105-110. doi: 10.1016/j.diabres.2016.03.018 | China | 82 | 1. Smart phone-based diabetes management application (Welltang)  2. Usual care control | NR |  |
|  | Zolfaghari, M., Mousavifar, S. A., Pedram, S., & Haghani, H. (2012). Journal of clinical nursing, 21(13‐14), 1922-1931. | Iran | 77 | 1. Nurse telephone follow up 2. SMS follow up | YES |  |

NK= not known; NR= not reported
